# Knowledge of pregnant women living in riverside community about exclusive breastfeeding in the context of Primary Health Care

**DOI:** 10.1590/1980-220X-REEUSP-2024-0361en

**Published:** 2025-06-20

**Authors:** Gabriela Tayane Santana do Espirito Santo, Jennifer Hillary Costa da Conceição Pompeu, Paulo Daniel Pereira Raad, Julia Oliveira Mendes, Erlon Gabriel Rego de Andrade, Eliene do Socorro da Silva Santos, Laura Maria Vidal Nogueira, Ivaneide Leal Ataíde Rodrigues

**Affiliations:** 1 Universidade do Estado do Pará, Escola de Enfermagem Magalhães Barata, Belém, PA, Brazil.; 2 Universidade do Estado do Pará, Escola de Enfermagem Magalhães Barata, Programa de Pós-Graduação em Enfermagem, Belém, PA, Brazil.

**Keywords:** Breast Feeding, Pregnant People, Rural Population, Rural Health, Primary Health Care

## Abstract

**Objective:**

To discuss the knowledge of pregnant women living in riverside communities about exclusive breastfeeding and its influence on the decision to breastfeed.

**Method:**

A descriptive and qualitative study, carried out with 20 pregnant women at the Municipal Health Unit of Cotijuba Island, in Belém, Pará, Brazil. Data were collected through semi-structured individual interviews, conducted between March and June 2024. The *corpus* was subjected to lexical analysis with *Interface de R pour les Analyses Multidimensionnelles de Textes et de Questionnaires* (0.7, alpha 2), using Descending Hierarchical Classification.

**Results:**

A total of 448 text segments were identified, of which 342 (76.34%) were used, generating six lexical classes, organized into two thematic axes, which presented the construction of knowledge about breastfeeding, mediated by social relations, and the knowledge that impacts the decision to breastfeed, highlighting the importance of breastfeeding for the growth and development of newborns and infants and the interface with complementary feeding.

**Conclusion:**

Despite challenging experiences and expectations and the discouragement caused by people around them, pregnant women considered breastfeeding as an opportunity to strengthen bonds with their children.

## INTRODUCTION

Exclusive breastfeeding (EBF) consists of exclusively offering breast milk to newborns and infants, which can occur through the breast or through ingestion of expressed milk, without adding any other type of food to the diet. It should be encouraged until the sixth month after birth, and it is recommended that it be maintained in a complementary way until the child is 2 years old. In addition to promoting adequate nutrition, impacting the growth and development of all organic systems and reducing infant mortality, EBF strengthens the bonds between mother and child, and contributes to preserving or improving maternal health^(1,2)^.

Considering the benefits of breast milk, the World Health Organization (WHO) expects that by 2030 at least 70% of children will be exclusively breastfed at the appropriate age^(3)^. In Brazil, although breastfeeding indicators have increased over the last few decades, the prevalence of EBF among children under 6 months of age was only 45.8%, according to data from the Brazilian National Survey on Child Nutrition (In Portuguese, *Estudo Nacional de Alimentação e Nutrição Infantil* – ENANI), published in 2021^(4)^.

Regardless of the distance from urban centers, traditional communities such as riverside populations are still in a vulnerable situation, characterized, among other factors, by low levels of education and lack of healthcare. In this socio-geographic context, governed by the dynamics of rivers and forests, pregnant women often face difficulties in accessing Primary Health Care (PHC) services, such as prenatal medical and nursing appointments, due to territorial and economic peculiarities that hinder their travel to health units^(5,6)^.

It is known that prenatal care is a favorable scenario for consolidating ideas and decisions related to EBF, including among pregnant women living in riverside communities, as it is during this period that they become familiar with the various demands that permeate the processes of gestation, birth and becoming a mother, in addition to understanding the importance of EBF^(7,8)^.

Populations whose main economic and subsistence activities are agriculture and fishing adopt diets that are related to such activities^(9)^. Traditional knowledge, shared between generations, can be effective in encouraging EBF, but it can also generate or strengthen myths about the subject, as it interferes with the decision to breastfeed, encourages early weaning and the inclusion of other foods before the recommended period^(10)^.

By investigating the knowledge of these pregnant women about EBF, it is possible to understand cultural, economic and social aspects that interfere with this practice and, thus, reflect on the articulation of these aspects with public policies and health programs that aim to meet health demands.

Given the relevance of the topic and the need to share it, the following guiding question was developed: what is the knowledge of pregnant women living in riverside community about EBF and how does it influence their decision to breastfeed? In order to answer this question, this study aimed to discuss the knowledge of pregnant women living in riverside community about EBF and its influence on their decision to breastfeed.

## METHOD

### Study Design

This is a descriptive and qualitative study, guided by the COnsolidated criteria for REporting Qualitative research (COREQ)^(11)^. It was decided to associate the descriptive nature with this approach, considering the possibilities that arise to characterize phenomena, including social ones, paying attention to subjective data, but also to aspects that particularize certain groups in relation to these phenomena^(12)^.

### Place

It was carried out at the Municipal Health Unit (MHU) of Cotijuba Island, located in the Administrative District of Outeiro, in the municipality of Belém, Pará, Brazil. Linked to the Municipal Health Department of Belém (In Portuguese, *Secretaria Municipal de Saúde de Belém* – SESMA), this unit offers services characteristic of PHC, such as prenatal appointments.

The island has around 10,000 inhabitants, and despite the urban transformations that resulted from factors such as proximity to other administrative districts of Belém and intensification of tourism activities, it still exhibits weaknesses in the provision of public policies, such as what occurs in local healthcare^(13,14)^. This setting was chosen because its geographic and sociocultural characteristics meet the interest of investigating the phenomenon of EBF in the context of a riverside population.

### Population and Selection Criteria

Twenty pregnant women from riverside community, aged 18 or over, regardless of gestational age and number of previous pregnancies, registered and undergoing regular prenatal care at the MHU, participated in the study. The minimum age was set at 18 years, considering that pregnancy in minors may involve legal issues or risk of emotional and psychological impairment for pregnant women. It was decided to exclude those who had physical and/or cognitive impairments that made it impossible to be interviewed. However, there were no exclusions or withdrawals; only two refused to participate due to unavailability to conduct an interview after three attempts.

During the data collection period, 22 pregnant women were eligible, resulting in 90.91% (20/22) participation. This meets the literature recommendation, which indicates 20 to 30 participants as the appropriate number to achieve the research objectives with a qualitative approach^(15)^.

### Data Collection

Initially, the researchers visited the MHU to introduce themselves to the professionals and inform them of the purpose and procedures of this research, at which time they learned about the dynamics of prenatal care and reserved a room with the manager to carry out the activities. Pregnant women were selected by convenience and approached personally, individually, before or after appointments, when they were invited to participate. To ensure comfort and privacy, those who agreed to participate were directed to a private room, where only participants and interviewers were present, who presented and clarified the details of the research in accessible language, with the aim of obtaining formal acceptance.

It is important to note that the unit professionals and pregnant women did not know the researchers in advance and, therefore, were not aware of their academic and professional trajectories or aspirations. Thus, it was decided to clarify all necessary aspects to the team and pregnant women, including the fact that the research comprised a set of extracurricular activities developed by four undergraduate nursing students of the Institutional Scientific Initiation Scholarship Program (In Portuguese, *Programa Institucional de Bolsas de Iniciação Científica* – PIBIC), two of whom were scholarship holders and two volunteers.

Data were collected between March and June 2024 through semi-structured individual interviews, using a script prepared by the researchers and consisting of two sections. The first ten questions addressed the sociodemographic and obstetric profile of participants, and variables on age, color/race, religion, education, occupation, monthly family income, people with whom they lived, marital status, number of pregnancies/children, and breastfeeding in previous pregnancies were investigated. The second part, with six subjective questions, explored the object of study, focusing on perceptions, experiences, and expectations surrounding EBF, addressed through dialogue.

In the script, subjective questions were worded as follows: 1) “What do you know about EBF?”; 2) “Do you think EBF is important for your child? Why?”; 3) “Do you intend to breastfeed your child? Why? If so, how will you do it?”; 4) “Do you talk to other people in your family (other women, partner) about EBF? Why? What do you talk about?”; 5) “During your prenatal appointments, did you receive any guidance about EBF? If so, what? How was this guidance shared? Who provided the guidance?”; 6) “Do you consider it important to receive information about EBF during prenatal care? If so, do you think this information influences your decision to breastfeed? Why?”. This instrument was not subjected to pilot testing, but it allowed sufficient understanding of the object, which is why a field diary was not used, no other collection techniques were incorporated and there was no need to repeat interviews. In order not to compromise the spontaneity of statements, it was decided not to share the transcripts with participants so that they could check and endorse them.

The interviews were conducted by four nursing students, one man and three women, divided into pairs and trained in orientation meetings, such as PIBIC activities and research group meetings linked to the authors’ institution, that mainly develops qualitative research. Since the interviews took place in two parts, corresponding to the script sections, the first part was manually recorded in the printed version of the instrument. Only the second part was audio-recorded in MP3 format, with an average duration of 15 minutes, sufficient to achieve the objective, compatible with other studies that used qualitative analysis techniques aided by the same software used in this study, which reported an average duration of 10 and 18 minutes^(16,17)^.

### Data Analysis and Processing

Sociodemographic and obstetric data were tabulated in Microsoft Office Excel^®^ (version 2013) and analyzed descriptively to highlight absolute and relative numbers. The interviews were transcribed to form a *corpus*, which was imported into *Interface de R pour les Analyses Multidimensionnelles de Textes et de Questionnaires* (IRaMuTeQ^®^, version 0.7, alpha 2) in a single, unformatted file (.txt file), with character encoding in the Unicode Transformation Format 8-bit (UTF 8) standard, in order to perform lexical analysis.

In compliance with the technical and methodological specificities of this software^(18)^, the 20 texts that constituted the *corpus* were individually identified by command lines formed by the following elements: four asterisks (****); blank space; asterisk next to the alphanumeric code assigned to the participant; blank space; nine sociodemographic and obstetric variables, identified by asterisk and specific codes (variable name abbreviation/acronym, underscore and number or abbreviation/capital letter/complementary acronym, according to the quantitative or qualitative nature of the variable), which were separated from each other by blank space and defined by the researchers as priorities, aiming not to lengthen command lines more than necessary.

These variables and their codes were: age (“Ag”, underscore and number corresponding to participants’ age); color/race (“CR”, underscore and abbreviation for color/race, with “Wh” for “white”, “Br” for “brown”, and “Bl” for “black”); education (“Ed”, underscore and acronym for education, with “IES” for “incomplete elementary school”, “IHS” for “incomplete high school”, “CHS” for “complete high school”, “VT” for “vocational training”, “IHE” for “incomplete higher education”, and “CHE” for “complete higher education”); religion (“Rel”, underscore and capital letter for religion, with “C” for “catholic”, “E” for “evangelical”, and “N” for “no religion”); marital status (“MS”, underscore and abbreviation or capital letter for status, with “Ma” for “married”, “S” for “single”, and “U” for “consensual union”); people with whom they lived (“PWL”, underscore and number of people); monthly family income (“FI”, underscore and number corresponding to the income in minimum wages, considering that, in Brazil, in 2024, the current wage was R$ 1,412.00, adding the word “less” or “more”, when necessary, to indicate that the income was lower or higher than the number informed); pregnancies (“P”, underscore and number); children (“C”, underscore and number). As an example of this organization, the following command line was assigned to the first participant: **** *P1 *Ag_29 *CR_Br *Ed_CHS *Rel_E *MS_S *PWL_2 *FI_1 *P_3 *C_2.

Frequently used in qualitative research and anchored in the R software functionalities, IRaMuTeQ^®^ was developed in 2009 by French scientist Pierre Ratinaud, with five analytical modalities to ensure the reliability of results: lexicographic analysis; specificities and correspondence factor analysis; Descending Hierarchical Classification (DHC); similarity analysis; and word cloud^(19,20)^.

In this study, DHC was used, through which the *corpus* was broken down into text segments (TSs) to generate lexical classes, according to the complementarity of the segments that constitute them, denoting meanings that individualize them, but that, together, express the phenomenon studied. These classes are illustrated by words arranged vertically in a dendrogram, to which IRaMuTeQ^®^ attributed, among other statistical values, the frequency (F) of TSs which presented each word in its respective class, and chi-square (X^2^), which demonstrates the associative strength of words, considering representative those that presented p < 0.0001. With this modality, the software identifies the best possibility to form classes that represent the *corpus* content, often resulting in the use of only a portion of the TSs, which must be at least 75% for the analysis to be considered reliable^(19,20)^.

The classes that resulted from subjective data analysis were organized into thematic axes, allowing their interpretation based on the pertinent and updated scientific literature. These axes and discussions related to them comprised a report sent to the authors’ institution, which chose not to share it with professionals from the unit or pregnant women, aiming to maintain its originality. Thus, they did not provide opinions on the results, but an ethical and technical-scientific commitment was made to forward the article(s) to professionals as soon as they were published and, if necessary, to schedule discussion groups to discuss the results, clarify doubts and discuss possible strategies that allow better intervention in the reality of pregnant women.

### Ethical Aspects

The study complied with Resolution 466/2012 of the Brazilian National Health Council/Ministry of Health, obtaining authorization from SESMA and approval from the Research Ethics Committee of the Undergraduate Nursing Course at the *Universidade do Estado do Pará*, under Opinion 6.631.984, issued in February 2024. All participants signed the Informed Consent Form. To maintain the confidentiality of their identities, an alphanumeric code consisting of the letter “P”, for “participant”, followed by a cardinal number, indicating the sequence of interviews, was used. Throughout the results, to identify the excerpts, it was decided to highlight both the alphanumeric codes and the command lines variables in parentheses.

## RESULTS

Participants’ age ranged from 19 to 37 years, with an average of 25.70 and a predominance of the age group from 19 to 23 (n = 9; 45%). Regarding skin color, 17 (85%) declared themselves to be brown, and as for religion, 11 (55%) were evangelical. Concerning education, 12 (60%) reported having completed high school, and regarding occupation, 11 (55%) had paid jobs and nine (45%) only performed household activities. Regarding monthly family income, ten (50%) declared up to one minimum wage. All of them lived with at least one person, and 12 (60%) were married or lived in a consensual union.

In relation to the number of pregnancies and children, nine (45%) were in their first pregnancy; six (30%) were in their second and had one child; four (20%) were in their third and had two children; one (5%) was in her fourth pregnancy and had three children; and 11 (55%) reported having breastfed in previous pregnancies.

The *corpus* entitled “Exclusive breastfeeding: knowledge of pregnant women living in riverside community in Primary Health Care” was broken down, resulting in the division of its 20 texts into 448 TSs and the identification of 15,674 occurrences (forms or words), with 2,149 distinct words and 1,132 hapaxes (words with a frequency equal to one), accounting for 7.22% of occurrences and 52.68% of distinct words. The average number of words per TS was approximately 34.99.

**Figure 1:**
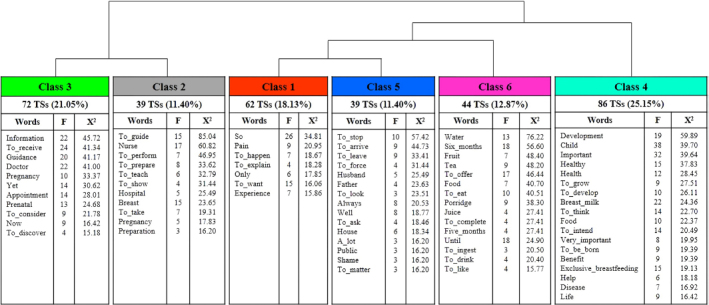
Descending Hierarchical Classification dendrogram. Belém, Pará, Brazil, 2024.

Through DHC, 342 TSs (76.34%) were used, structuring two *subcorpora*, with six lexical classes: the first *subcorpus*, formed by classes 2 and 3, and the second, by classes 4, 6, 1 and 5, according to the *corpus* partition logic (Figure 1). The secondary subjective data that constituted the unused percentage of the *corpus* (23.66%) were not considered.

These classes were organized into two thematic axes, associated respectively with the social construction of knowledge about breastfeeding and the knowledge that impacts the decision to breastfeed, which is why the authors gave them titles that allude to these meanings.

Composing the first axis, classes 3, 2, 1 and 5 were called “Importance of information about breastfeeding in prenatal care”, “Nurses’ guidelines on breastfeeding”, “Experiences and expectations about the act of breastfeeding” and “Dialogues with different subjects in daily life”. In the second axis, classes 4 and 6 were given the titles “Importance of exclusive breastfeeding for growth and development” and “Challenges and possibilities of complementary feeding”. Both are presented below, highlighting their classes and some emblematic excerpts.

### Thematic axis 1 – Social construction of knowledge about breastfeeding (classes 3, 2, 1 and 5)

In class 3, 72 TSs (21.05% of the *corpus*) and 11 representative words (p < 0.0001) were identified, indicating perceptions that highlight the importance of guidance provided by healthcare professionals in prenatal appointments (Figure 1).

Words such as “information” (X^2^ = 45.72), “to_receive” (X^2^ = 41.34), “guidance” (X^2^ = 41.17) and “appointment” (X^2^ = 28.01) represented this characteristic, indicating the sharing of information in the formal environment of the MHU. In this sense, participants understood medical and nursing guidelines as reliable and safe tools, highlighting that sharing them can contribute to better informing them:

*I received this information before giving birth, because the nurse gave lectures to pregnant women. That was when I acquired a little knowledge and I was intrigued, because grandma said one thing and mom said another.* (P10, Ag_22, CR_Br, Ed_VT, Rel_E, MS_U, PWL_2, FI_more_1, P_2, C_1)

*Today was my first prenatal appointment, so I haven’t received any information about exclusive breastfeeding yet. However, I think it’s important to receive this information, including at the first appointment, during this first contact with professionals, because I’m informed, since it’s something I don’t know yet.* (P14, Ag_22, CR_Br, Ed_CHS, Rel_C, MS_S, PWL_2, FI_1, P_1, C_0)

Composed of 39 TSs (11.40% of the *corpus*) and 11 representative words (p < 0.0001), class 2 revealed how guidance from professionals, especially nurses, can strengthen the construction of knowledge about breastfeeding. The word “nurse” (X^2^ = 60.82), which presented the second highest chi-square of the class, associated with the verbs “to_guide” (X^2^ = 85.04) and “to_teach” (X^2^ = 32.79), expresses that participants recognized having access, in the current pregnancy and/or in a previous pregnancy, to guidance provided by nurses in different healthcare services, such as in the MHU:

*The nurse gave guidance on the use of diaper rash ointment and teas, because she was not in favor of offering these* [to the child]. *She also talked about the importance of breastfeeding and that not every time the child cried it was time to breastfeed. We had to learn to understand when it was time to breastfeed and when the child was in pain.* (P10, Ag_22, CR_Br, Ed_VT, Rel_E, MS_U, PWL_2, FI_more_1, P_2, C_1)

*During prenatal care, the nurse always advised me on how to take care of my breasts. She advised me to expose myself to the sun, stimulate my nipples while bathing, not rub soap on my nipples too often to avoid drying them out, and, if necessary, to go shirtless to get the sunlight vitamin.* (P12, Ag_29, CR_Wh, Ed_CHS, Rel_E, MS_U, PWL_3, FI_1, P_3, C_2)

Although they stated that they were guided during the gestational period, it was noted that the guidance did not sufficiently clarify the aspects that permeate the topic. The words “breast” (X^2^ = 23.65) and “pregnancy” (X^2^ = 17.83), associated with the verbs “to_perform” (X^2^ = 46.95), “to_prepare” (X^2^ = 33.62) and “to_take” (X^2^ = 19.31), demonstrate that, even so, some participants had a vague idea of ​​how to prepare their breasts for the act of breastfeeding, either due to the lack of guidance or due to a certain imprecision or incoherence in some reports:

*There needs to be counseling, at least the basics, which is really not being done. I have already had four prenatal appointments; I ask questions to healthcare professionals, but they just say that I have to moisturize my breasts, that’s all.* (P9, Ag_22, CR_Br, Ed_IHE, Rel_N, MS_Ma, PWL_1, FI_1, P_1, C_0)

*During prenatal appointments, people ask me if I prepare my breasts for breastfeeding. They say it’s good to prepare them, but I say I don’t prepare them because they told me at the hospital that doing so wasn’t good.* (P15, Ag_21, CR_Br, Ed_IHS, Rel_E, MS_U, PWL_2, FI_1, P_2, C_1)

Comprising 62 TSs (18.13% of the *corpus*) and seven representative words (p < 0.0001), class 1 was characterized by words such as “pain” (X^2^ = 20.95), “to_want” (X^2^ = 16.06) and “experience” (X^2^ = 15.86), addressing some repercussions of breastfeeding in the daily lives of pregnant women who have already experienced this act as nursing mothers or who expected to breastfeed. It was identified that breastfeeding is a process that can generate many expectations and experiences, including negative ones, given the physical sensations and feelings related to it.

Some TSs have shown that the act of breastfeeding can cause pain and discomfort in women, even those who are not primiparous:

*I believe that every pregnant woman goes through this, even those who are not first-time mothers. Even those who are having their second child will suffer from pain when breastfeeding.* (P11, Ag_22, CR_Br, Ed_IHE, Rel_E, MS_S, PWL_11, FI_1, P_1, C_0)

*Many women complain about breastfeeding; they say that their breasts become saggy and that they feel pain. For example, my cousin said that she felt a lot of pain, so I was scared and that influences me, but each woman has her own experience, each woman is unique.* (P14, Ag_22, CR_Br, Ed_CHS, Rel_C, MS_S, PWL_2, FI_1, P_1, C_0)

In other reports, participants expressed fear and uncertainty, perhaps because they were unable to breastfeed, with negative repercussions on their children’s growth and development:

*After ten years, having to go through all that again* [difficulties breastfeeding the first child], *I am uncertain about breastfeeding.* (P4, Ag_32, CR_Br, Ed_CHS, Rel_E, MS_Ma, PWL_2, FI_1, P_2, C_1)

*I’m afraid of creating expectations about wanting to breastfeed my son, and I know that I might not be able to. So, I’m afraid of getting frustrated, disappointed and not being able to breastfeed.* (P9, Ag_22, CR_Br, Ed_IHE, Rel_N, MS_Ma, PWL_1, FI_1, P_1, C_0)

Despite the negative feelings and feelings, as highlighted in the four previous excerpts, it was identified that pregnancy can also contribute to forming bonds between mother and child, built throughout the weeks of pregnancy and strengthened after birth:

*I believe that exclusive breastfeeding will help me create a bond with my baby; there will be a connection between us. With physical contact, he will feel welcomed through breastfeeding.* (P6, Ag_24, CR_Bl, Ed_CHS, Rel_N, MS_Ma, PWL_2, FI_more_1, P_1, C_0)

*When my son latched on for the first time* [referring to the first breastfeeding], *I cried because I had a very different feeling, it was something I can’t explain, a strength unlike anything else in my breasts. Something happened, a loving bond! From then on, I was sure that I would have to breastfeed not for me, but for him.* (P10, Ag_22, CR_Br, Ed_VT, Rel_E, MS_U, PWL_2, FI_more_1, P_2, C_1)

Class 5 corresponds to 39 TSs (11.40% of the *corpus*), which is why classes 2 and 5 are the smallest, with 15 representative words (p < 0.0001), characterized by verbs such as “to_stop” (X^2^ = 57.42), “to_leave” (X^2^ = 33.41), “to_force” (X^2^ = 31.44) and “to_look” (X^2^ = 23.51), in addition to other terms, such as “husband” (X^2^ = 25.49), “public” (X^2^ = 16.20) and “shame” (X^2^ = 16.20). Its content addresses the dialogues that pregnant women developed on the topic with various social subjects, such as family members, healthcare professionals, neighbors and even strangers. Based on the reports, these interactions had a significant impact on women’s perceptions, generating insecurity in some of them, depending on the information shared.

Some healthcare professionals, who should offer support and appropriate guidance, have sometimes advised against breastfeeding and pressured for its cessation, making it a stressful and unsafe experience:

*People put a lot of pressure on me to stop breastfeeding; they said that my son was already big. Once, when I arrived at the* [health] *unit with him sick, the doctor who saw me insisted that I stop breastfeeding; he said that there was no need and that the calf only nurses for I don’t know how many weeks.* (P10, Ag_22, CR_Br, Ed_VT, Rel_E, MS_U, PWL_2, FI_more_1, P_2, C_1)

[...] *at that moment, there was a clash; I wouldn’t stop breastfeeding my son just because someone was bothered, I’m not forced to do that. It’s my right to breastfeed!* (P12, Ag_29, CR_Wh, Ed_CHS, Rel_E, MS_U, PWL_3, FI_1, P_3, C_2)

Although breastfeeding is a natural and essential process for life, some reports highlighted conflicts regarding its performance in public spaces, in which some people, often strangers, claimed discomfort when witnessing it, attributing a certain shame to mothers:

*People would look at my son and tell him that he was too big, that he should be ashamed and stop breastfeeding. Sometimes I would feel ashamed, ashamed and cornered.* (P10, Ag_22, CR_Br, Ed_VT, Rel_E, MS_U, PWL_2, FI_more_1, P_2, C_1)

*I have had conflicting situations regarding breastfeeding. They asked me if I wasn’t ashamed to breastfeed in public; they said they were uncomfortable watching me breastfeed and that they didn’t have to watch that.* (P12, Ag_29, CR_Wh, Ed_CHS, Rel_E, MS_U, PWL_3, FI_1, P_3, C_2)

Even in the face of discouraging dialogues, disrespectful situations and social pressures on certain occasions, including from family members, women persisted in breastfeeding or declared their intention to breastfeed, recognizing its importance:

*My mother said that I was getting ugly* [from breastfeeding so much] *and that my husband would change me, but that’s up to him. If it’s not meant to be mine, it won’t be mine, but I’m not going to stop breastfeeding.* (P10, Ag_22, CR_Br, Ed_VT, Rel_E, MS_U, PWL_2, FI_more_1, P_2, C_1)

*My husband says that she* [sister-in-law] *and I are alike because we are not ashamed. If people talk, I don’t care; I will breastfeed my child first. Other people should stop caring.* (P16, Ag_27, CR_Br, Ed_IHS, Rel_C, MS_S, PWL_4, FI_1, P_4, C_3)

### Thematic axis 2 – Knowledge that impacts the decision to breastfeed (classes 4 and 6)

Composed of 86 TSs (25.15% of the *corpus*), class 4 is the largest and presents 18 representative words (p < 0.0001), such as “development” (X^2^ = 59.89), “important” (X^2^ = 39.64), “health” (X^2^ = 28.45), “to_grow” (X^2^ = 27.51), “to_develop” (X^2^ = 26.11), “breast_milk” (X^2^ = 24.36), “food” (X^2^ = 22.37), “benefit” (X^2^ = 19.39) and “life” (X^2^ = 16.42). Associated with the repercussions of EBF on children’s bodies, these terms demonstrate that participants characterized breastfeeding as a practice that contributes to the healthy growth and development of their children, which is why they decided to breastfeed, by relating breast milk to strengthening children’s immunity and reducing the chances of illness:

*I believe that it develops everything, cells, growth, having antibodies to* [fight] *diseases and viruses. I believe that breastfeeding contributes positively to my two children.* (P12, Ag_29, CR_Wh, Ed_CHS, Rel_E, MS_U, PWL_3, FI_1, P_3, C_2)

*The first six months of breastfeeding are important for the child’s overall development, and this will be good for preventing future illnesses, making the child strong.* (P13, Ag_37, CR_Br, Ed_CHE, Rel_N, MS_U, PWL_1, FI_more_2, P_1, C_0)

However, they reported that there are differences in breast milk and that some women have “weaker” milk, which does not contribute to the child’s growth and overall development, while “strong” milk is considered more nutritious and responsible for adequate growth and development. They also highlighted that milk production is related to maternal nutrition, as they believe that nutrition is a source of nutrients, associated with the quantity and quality of milk:

*They told me that I should have a healthy diet. The more I ate right, the more breast milk I would produce, because, in addition to taking care of myself and preparing myself, we give them* [the children] *a better life.* (P1, Ag_29, CR_Br, Ed_CHS, Rel_E, MS_S, PWL_2, FI_1, P_3, C_2)

*I believe that* [breast milk] *is very important, because it is his* [my child’s] *food. I know that my diet will improve the milk and provide health for him.* (P6, Ag_24, CR_Bl, Ed_CHS, Rel_N, MS_Ma, PWL_2, FI_more_1, P_1, C_0)

Comprising 44 TSs (12.87% of the *corpus*) and 15 representative words (p < 0.0001), class 6 reveals that pregnant women were aware that EBF should be carried out exclusively with breast milk during the first six months after birth and, after this period, other foods should be offered to complement breastfeeding. The term “six_months” (X^2^ = 56.60) presented the second highest chi-square of the class, associated with the words “to_complete” (X^2^ = 27.41) and “until” (X^2^ = 24.90):

*Exclusive breastfeeding is when I breastfeed the child until six months. The doctor says it’s about the child’s well-being. The child can’t drink water, eat or anything like that, because they could get sick.* (P3, Ag_35, CR_Br, Ed_IES, Rel_E, MS_S, PWL_2, FI_1, P_3, C_2)

*I’m not going to offer fruit to my one-month-old son, because he won’t know how to eat an apple, he won’t have the practice of chewing or swallowing something that is harder.* (P9, Ag_22, CR_Br, Ed_IHE, Rel_N, MS_Ma, PWL_1, FI_1, P_1, C_0)

The words “water” (X^2^ = 76.22), “fruit” (X^2^ = 48.40), “tea” (X^2^ = 48.20), “food” (X^2^ = 40.70), “porridge” (X^2^ = 38.30) and “juice” (X^2^ = 27.41) express that some factors can interfere in the decision to breastfeed exclusively. Challenges related to the cultural aspects that permeate breastfeeding, such as the belief that breast milk may not satisfy the child, encouraged the planning of another food source as an alternative. Moreover, the need to return to occupational activities stood out as a relevant socioeconomic aspect, as some participants pointed out that it can interfere with EBF and harm the bonds between mother and child, increasing the possibility of introducing other foods early. These aspects were indicated, for instance, in the statements of P8 and P3, respectively:

*I will offer porridge and breast milk. If he is not satisfied with milk, I will offer porridge in the morning, at least once a day.* (P8, Ag_21, CR_Br, Ed_CHS, Rel_C, MS_U, PWL_3, FI_less_1, P_1, C_0)

*I’m going to breastfeed for at least four or five months. I don’t plan on breastfeeding until six months, because my daughter was very attached, she didn’t want to be with anyone and I couldn’t work.* (P3, Ag_35, CR_Br, Ed_IES, Rel_E, MS_S, PWL_2, FI_1, P_3, C_2)

## DISCUSSION

In the first thematic axis, the social construction of knowledge about breastfeeding highlighted the importance of sharing information about EBF, through pregnant women’s perceptions about the guidance they received from healthcare professionals, a context in which the dialogues with these subjects pointed to a process of clarification and strengthening of maternal knowledge, making it possible to infer that the socially constructed information had a favorable impact on the decision to breastfeed. However, during prenatal care, insufficient communication between pregnant women and professionals can generate uncertainty and conflicts related to this decision.

During pregnancy and childbirth, guidance should be based on pregnant women’s particularities, considering cultural, economic, social and territorial aspects, aiming to promote a healthy pregnancy and encourage EBF. These women need professional support and care based on comprehensiveness and longitudinality, in addition to educational processes that are not merely explanatory and that value their concerns. PHC is a crucial setting in this context, representing the main level of care accessed by pregnant women and infants seeking information and perinatal care, and is the space where they should be supported to strengthen their knowledge and monitor breastfeeding^(21,22)^.

Professionals who share information about EBF and complementary information with pregnant women and their families early on act as agents who promote protective mechanisms for breastfeeding. EBF is directly related to the frequency of pregnant women’s prenatal appointments, since the more they attend healthcare services during this period, the greater the possibilities for discussing the topic. Research conducted in the state of Bahia revealed social and regional inequities as relevant conditions that interfere with the quality of perinatal care, concluding that pregnant women in northeastern Brazil still have lower coverage of PHC services, when compared to those in southern Brazil, which have higher EBF indicators, possibly due to the better care they receive in PHC^(2)^.

Healthcare conditions for pregnant women living in riverside communities in the Amazon region are similar to those of pregnant women in Bahia, since, historically, the North and Northeast regions have presented many challenges related to healthcare services and peculiar cultural aspects, which must be considered in care activities^(2,23)^. The importance of nurses was also noted, as their guidance can reinforce the act of breastfeeding and consolidate pregnant women’s knowledge. These guidelines were materialized in clarifications about the particularities of breastfeeding, demonstrating participants’ previous conceptions about EBF, although the knowledge of some was technically more structured and organized than that of others.

The literature addresses how health education activities promoted by nurses can boost pregnant women’s knowledge, resulting in good breastfeeding performance, since, with clarifications and accessible language, technical-scientific information can be shared appropriately, generating comfort and security^(1,24)^. Explanations about breast care, the body position for the child to be breastfed and breastfeeding duration, among other aspects, can positively influence EBF and prevent its premature interruption^(25)^.

Nurses are an active agent of care who approach users and create bonds, recognizing the biopsychosocial characteristics of each pregnant woman as a necessary requirement for personalized care. This must occur through skills and abilities developed throughout academic training and professional practice, with which they perform clinical examination (anamnesis and physical examination), incorporated into sensitive listening and the necessary conduct to solve or avoid problems^(26)^.

Due to the social determinants of riverside populations’ health and failures often present in PHC services, there are obstacles that limit personalized care, compromising its effectiveness. Due to a lack or shortage of qualified professionals, the conditions of several communities require additional efforts from nurses to share information about EBF and meet other demands^(5)^. In this regard, the use of educational technologies, with accessible verbal and/or non-verbal language, can facilitate guidance on the topic, demystifying outdated aspects that may interfere with the decision to breastfeed^(25)^.

In the knowledge construction, experiences and expectations were revealed, which is why, in light of results, it is understood that it is essential to know maternal experiences, since, in certain cases, breastfeeding can generate insecurity and have a negative impact on the way women represent it, encouraging early weaning^(27)^.

The concerns that some women expressed about the possibility of not being able to breastfeed and the pain that breastfeeding can cause in their breasts were due to a lack of experience, negative experiences or the sharing of knowledge with other women. Physiologically, breast pain results from breast engorgement or injuries to these organs, with the main cause being the inadequate body positioning of the child or mother when breastfeeding, which can interrupt breast milk production due to sucking stimuli reduction, weakening the possibilities of maintaining breastfeeding^(28)^. Despite this, pregnant women understood breastfeeding as a process that connects its leading actors (mother and child), strengthening the bonds between them. It can be inferred that such understanding is capable of motivating EBF, because, as knowledge is established, there is encouragement for them to breastfeed.

Pregnant women’s perceptions were significantly influenced by experiences of dialogue with subjects in their daily lives, such as family members, healthcare professionals, neighbors and others. On certain occasions, social interactions were positive and, on others, negative, since they allowed them to experience some situations of encouragement for EBF, but also discouraging attitudes. In light of literature, this fact may have repercussions on the biological and psychosocial dimensions of pregnant women^(29)^, since a portion of reports demonstrated shame and lack of motivation to continue EBF.

Since it is a social process, support networks are essential for breastfeeding, as social interactions strongly influence its implementation and continuity. The knowledge and social practices surrounding EBF are heterogeneous and influenced by these interactions, due to the social context and role played by women in the community. Thus, EBF may or may not be successful, depending on this role and other factors, such as previous experiences, guidance on the subject, and family and social support^(10,21)^. In family relationships, there is a historical and sociocultural process characterized by the intergenerational sharing of knowledge about breastfeeding, led by individuals with notable representation, such as mothers and grandmothers. Generally, there is trust in the guidance of family members, due to the coexistence and understanding of belonging by pregnant women^(21)^.

Regarding the second thematic axis, the knowledge that affects the decision to breastfeed revealed pregnant women’s understanding of the repercussions of breast milk on the child’s body, which was revealed through the symbolic association of EBF with the figure of a healthy and physically well-developed child. They also expressed the idea that mothers’ diet influences breast milk production, as their nutritional condition determines whether this food is sufficient or insufficient to meet the child’s nutritional demands.

In addition to bonding, EBF represents one of the main ways to promote health, offering other benefits in the short and long term, as it has important effects on the development of oral muscles, since it requires sucking movements that involve the simultaneous activity of different muscles. It also reduces infant mortality and the occurrence of infections, allergies and obesity, in addition to promoting the release of oxytocin, a hormone that stimulates feelings of pleasure and well-being, acting on breast milk secretion, a rich source of nutrients and antibodies, being the most complete and effective food to nourish the newborn, as it strengthens its immune system^(7,30)^.

For mothers, breastfeeding enables rapid recovery in the postpartum period, reducing the risk of bleeding and the occurrence of anemia, breast cancer and ovarian cancer^(27,30)^. Its benefits for the child’s psychological development also stand out, culminating in representing, together with other benefits, a sustainable alternative for maintaining or recovering the balance of family income^(8)^.

One of the main myths of common sense is the idea that breast milk is weak and insufficient to nourish the child. This consideration is made considering that, due to its completeness, this food has all the organic and inorganic compounds necessary for the maintenance of the body in the first six months after birth, such as carbohydrates, lipids, proteins, water and mineral salts^(5,31)^. Its constitution is influenced by mothers’ diet, which is why it is necessary to take care of the diet so that it is produced with better quality^(31)^. In this process, aspects that should stand out the most are adherence to EBF, self-care and women’s autonomy during its implementation^(21,29)^.

Pregnant women expressed the importance of breastfeeding exclusively for the first six months and, after that, introducing other foods as a complement. The literature shows that some factors can reinforce this understanding and corresponding maternal practices, among which are regular monitoring of growth and development, through childcare appointments, especially in PHC units, and guidance provided during prenatal care by qualified professionals who ensure ongoing clarification, with information on the nutritional sufficiency of breast milk^(27,32)^.

However, participants reported some barriers that must be overcome for EBF to be effective, with an emphasis on cultural aspects, since third-party beliefs, when shared with pregnant women, can influence their decision to breastfeed. Within the riverside context, cultural determinants can promote women’s willingness to offer other foods early^(33)^, as evidenced by reports that certain foods supposedly cause satiety when compared to breast milk. Given the social vulnerabilities that circulate among riverside populations, education is an important factor, as it has an impact on this decision, given that early introduction of food tends to be reduced when there is proper understanding of the importance of EBF and the introduction of other foods at an appropriate time^(34)^.

It is also worth highlighting socioeconomic aspects that, together with cultural ones, encouraged early complementary feeding, as some women intended to offer other foods before completing the sixth month, due to the need to return to work. In line with this result, evidence shows that the higher the socioeconomic level of pregnant women, the lower the chance of starting complementary feeding before this period^(34,35)^.

The results also highlighted a set of challenges related to EBF that are inherent to various human groups, as they are socially determined by factors such as maternal education and occupation, family income and support, guidance and other types of care provided by healthcare services, pointing to weaknesses that must be overcome in maternal and child healthcare in these groups’ daily lives, as demonstrated in a broad scoping review, which included 145 studies carried out in Africa, Asia, the Americas and the Middle East^(36)^.

This does not invalidate or contradict the fact that riverside populations are endowed with specificities that arise from the reality they experience and their ways of life governed by the dynamics of rivers and forests, a context in which the processes of gestation, birth, becoming a mother and breastfeeding imply many collective challenges, as reiterated in the literature^(5,7,37,38)^. However, considering EBF as a social phenomenon, it is necessary that other studies be carried out with riverside populations in different scenarios to produce evidence that points out aspects more directly related to the specificities of these populations, highlighting their differences in relation to others. The results revealed here can support the design of new objects to be investigated.

As a limitation of this study, it is mentioned that it was carried out in a health unit with riverside pregnant women living in a specific setting in the municipality of Belém, configuring less geographic representativeness of the data. For this reason, it is considered that the knowledge on the subject was influenced by cultural, socioeconomic and territorial characteristics, preventing it from being generalized more broadly, although it presents possible similarities with the realities of other riverside populations in the local, regional and national scenarios.

Thus, the study contributes to advances in nursing and public health, as it can foster spaces for discussion and reflection among students and professionals who work in assisting these populations, management and administration of healthcare services, in teaching and research.

## CONCLUSION

This study revealed the knowledge of pregnant women living in riverside community about EBF in PHC, demonstrating how it influenced the decision to breastfeed. Qualified information on the topic was considered essential, especially when shared by nurses and other professionals in prenatal care, given that some information was insufficient and generated insecurity and doubts among pregnant women.

Despite challenging experiences and expectations and discouragement from people around them, pregnant women attributed breastfeeding as an opportunity to strengthen bonds with their children. They also highlighted the importance of breast milk for the growth and development of children, although at the same time they highlighted cultural and socioeconomic aspects that influenced their decision-making.

Considering the importance of PHC in meeting human groups’ health needs, it is understood that such results can support strategies to promote riverside populations’ health and encourage EBF with culturally appropriate information and feasible practices, especially in the Amazon context, valuing the diversity and particularities of these populations.

To this end, it is necessary to develop and implement, in higher education and continuing education, teaching and learning strategies that develop the cultural competency of human resources in the health field, with the aim of inducing training processes aligned with groups’ needs with their own characteristics and that require differentiated interventions, as is the case of riverside populations. This consideration is made to emphasize that universality, comprehensiveness and equity, doctrinal principles of the Brazilian Health System, must be implemented in care practices.

Strategies that allow this development are particularly necessary in nursing professionals’ daily lives, especially nurses, whose work processes inherent to care practices, management, teaching, research and political participation demand a high sense of scientific, ethical, technical and social responsibility so that the conscious and safe exercise of the profession mobilizes transformations amid the challenges of the scenarios where riverside populations live.

## Data Availability

The data supporting this study are available upon request to the corresponding author.
