# Supplementary material for: Knowledge of pregnant women living in riverside community about exclusive breastfeeding in the context of Primary Health Care
Source: Rev Esc Enferm USP. 2025 Jun 20;59:e20240361. doi: 10.1590/1980-220X-REEUSP-2024-0361en (PMC12184773; doi:10.1590/1980-220X-REEUSP-2024-0361en)
Supplement: Translation

# Translation: Saberes de gestantes ribeirinhas sobre o aleitamento materno exclusivo no contexto da Atenção Primária à Saúde

**DOI:** 10.1590/1980-220X-REEUSP-2024-0361pt

**Authors:** Gabriela Tayane Santana do Espirito Santo, Jennifer Hillary Costa da Conceição Pompeu, Paulo Daniel Pereira Raad, Julia Oliveira Mendes, Erlon Gabriel Rego de Andrade, Eliene do Socorro da Silva Santos, Laura Maria Vidal Nogueira, Ivaneide Leal Ataíde Rodrigues

**Affiliations:** 1 Universidade do Estado do Pará, Escola de Enfermagem Magalhães Barata, Belém, PA, Brasil.; 2 Universidade do Estado do Pará, Escola de Enfermagem Magalhães Barata, Programa de Pós-Graduação em Enfermagem, Belém, PA, Brasil.

**Keywords:** Aleitamento Materno, Gestantes, População Rural, Saúde da População Rural, Atenção Primária à Saúde

## Abstract

**Objetivo:**

Discutir os saberes de gestantes ribeirinhas sobre o aleitamento materno exclusivo e sua influência na decisão de amamentar.

**Método:**

Estudo descritivo e qualitativo, realizado com 20 gestantes na Unidade Municipal de Saúde da Ilha de Cotijuba, em Belém, Pará, Brasil. Os dados foram coletados por entrevistas individuais semiestruturadas, realizadas entre março e junho de 2024. O *corpus* foi submetido à análise lexical com o *software Interface de R pour les Analyses Multidimensionnelles de Textes et de Questionnaires* (0.7, *alpha* 2), empregando a Classificação Hierárquica Descendente.

**Resultados:**

Identificaram-se 448 segmentos de texto, dos quais foram aproveitados 342 (76,34%), gerando seis classes lexicais, organizadas em dois eixos temáticos, que apresentaram a construção dos saberes sobre amamentação, mediada por relações sociais, e os saberes que repercutem na decisão de amamentar, evidenciando a importância do aleitamento para o crescimento e o desenvolvimento de recém-nascidos e lactentes e a interface com a alimentação complementar.

**Conclusão:**

Apesar das vivências e expectativas desafiadoras e do desencorajamento suscitado por pessoas de seu convívio, as gestantes zelavam pela amamentação como oportunidade para fortalecer os vínculos com seus filhos.

## INTRODUÇÃO

O aleitamento materno exclusivo (AME) consiste na oferta exclusiva de leite materno aos recém-nascidos e lactentes, podendo ocorrer através da mama ou ingestão de leite ordenhado, sem que qualquer outro tipo de alimento seja acrescentado à dieta. Deve ser incentivado até o sexto mês após o nascimento, recomendando-se que seja mantido de forma complementar até a criança completar dois anos de idade. Além de promover a nutrição adequada, repercutindo no crescimento e no desenvolvimento de todos os sistemas orgânicos e na redução da mortalidade infantil, o AME fortalece os vínculos entre mãe e filho, e contribui para preservar ou aumentar a saúde materna^(1,2)^.

Considerando os benefícios do leite materno, a expectativa da Organização Mundial da Saúde (OMS) é que até 2030 pelo menos 70% de crianças sejam amamentadas de forma exclusiva na idade oportuna^(3)^. No Brasil, embora os indicadores de amamentação tenham crescido ao longo das últimas décadas, a prevalência de AME entre crianças com idade inferior a seis meses foi de apenas 45,8%, segundo dados do Estudo Nacional de Alimentação e Nutrição Infantil (ENANI), publicado em 2021^(4)^.

Independentemente da distância em relação aos centros urbanos, comunidades tradicionais, como as populações ribeirinhas, ainda se encontram em situação de vulnerabilidade, caracterizada, entre outros fatores, por baixos níveis de escolaridade e falta de assistência à saúde. Nesse contexto sociogeográfico, regido pela dinâmica dos rios e das florestas, muitas vezes, as gestantes enfrentam dificuldades para acessar os serviços da Atenção Primária à Saúde (APS), a exemplo das consultas médicas e de enfermagem no pré-natal, devido às peculiaridades territoriais e econômicas que obstaculizam seu deslocamento às unidades de saúde^(5,6)^.

Sabe-se que o pré-natal é um cenário favorável para consolidar ideias e decisões relacionadas ao AME, inclusive entre gestantes ribeirinhas, pois é nesse período que elas conhecem várias demandas que permeiam os processos de gestar, parir e tornar-se mãe, além de conhecer a importância do AME^(7,8)^.

Populações que têm como principais atividades econômicas e de subsistência a agricultura e a pesca adotam dietas que guardam relação com tais atividades^(9)^. Os saberes tradicionais, compartilhados entre gerações, podem ser eficazes para incentivar o AME, mas também gerar ou fortalecer mitos acerca do tema, na medida em que interferem na decisão de amamentar, incentivam o desmame precoce e a inclusão de outros alimentos antes do período recomendado^(10)^.

Ao investigar os saberes dessas gestantes acerca do AME, pode-se compreender aspectos culturais, econômicos e sociais que interferem nessa prática e, assim, refletir sobre a articulação desses aspectos com políticas públicas e programas de saúde que visam atender às demandas de saúde.

Diante da relevância do tema e da necessidade de compartilhá-lo, elaborou-se a seguinte questão norteadora: quais são os saberes de gestantes ribeirinhas sobre o AME e como influenciam a decisão dessas mulheres para amamentar? No intuito de responder a essa questão, este estudo objetivou discutir os saberes de gestantes ribeirinhas sobre o AME e sua influência na decisão de amamentar.

## MÉTODO

### Tipo de Estudo

Estudo descritivo e qualitativo, guiado pelo *COnsolidated criteria for REporting Qualitative research* (COREQ)^(11)^. Optou-se por associar a natureza descritiva com essa abordagem, considerando as possibilidades que se apresentam para caracterizar fenômenos, incluindo os sociais, atentando para os dados subjetivos, mas também para os aspectos que particularizam determinados grupos em relação a esses fenômenos^(12)^.

### Local

Foi realizado na Unidade Municipal de Saúde (UMS) da Ilha de Cotijuba, localizada no Distrito Administrativo de Outeiro, no município de Belém, Pará, Brasil. Vinculada à Secretaria Municipal de Saúde de Belém (SESMA), essa unidade oferece serviços característicos da APS, como consultas de pré-natal.

A Ilha tem cerca de 10.000 habitantes, e apesar das transformações urbanas que resultaram de fatores como a proximidade em relação a outros distritos administrativos de Belém e a intensificação das atividades de turismo, ainda exibe fragilidades na oferta de políticas públicas, a exemplo do que ocorre na atenção à saúde local^(13,14)^. Esse cenário foi escolhido por suas características geográficas e socioculturais atenderem ao interesse de investigar o fenômeno do AME no contexto de uma população ribeirinha.

### População e Critérios de Seleção

Participaram 20 gestantes ribeirinhas, com idade igual ou superior a 18 anos, independentemente da idade gestacional e do número de gestações anteriores, cadastradas e em acompanhamento pré-natal regular na UMS. Definiu-se a idade mínima de 18 anos, considerando que a gravidez em menores pode envolver aspectos legais ou risco de comprometimento emocional e psíquico para a gestante. Optou-se por excluir as que apresentavam comprometimento físico e/ou cognitivo que impossibilitasse a entrevista. Todavia, não houve exclusões ou desistências; apenas duas recusaram participar por indisponibilidade para realizar entrevista após três tentativas.

No período de coleta dos dados, 22 gestantes eram elegíveis, obtendo-se 90,91% (20/22) de participação. Isso atende à recomendação da literatura, que indica de 20 a 30 participantes como número adequado para alcançar os objetivos de pesquisa com abordagem qualitativa^(15)^.

### Coleta dos Dados

Inicialmente, os pesquisadores visitaram a UMS para se apresentar aos profissionais e deixá-los cientes da finalidade e dos procedimentos da pesquisa, oportunidade em que conheceram a dinâmica do acompanhamento pré-natal e reservaram com o gerente uma sala para desenvolver as atividades. As gestantes foram selecionadas por conveniência e abordadas pessoalmente, de maneira individual, antes ou depois dos atendimentos, quando foram convidadas a participar. Para garantir conforto e privacidade, as que aceitaram participar foram encaminhadas à sala reservada, onde estavam apenas a participante e os entrevistadores, que apresentaram e esclareceram os detalhes da pesquisa com linguagem acessível, no intuito de obter o aceite formal.

Ressalta-se que os profissionais da unidade e as gestantes não conheciam antecipadamente os pesquisadores e, portanto, não estavam a par de suas trajetórias ou aspirações acadêmicas e profissionais. Assim, optou-se por esclarecer todos os aspectos necessários à equipe e às gestantes, incluindo o fato de que a pesquisa compunha um conjunto de atividades extracurriculares desenvolvido por quatro estudantes de graduação em enfermagem do Programa Institucional de Bolsas de Iniciação Científica (PIBIC), sendo dois bolsistas e dois voluntários.

Os dados foram coletados entre março e junho de 2024, por meio de entrevistas individuais semiestruturadas, mediante roteiro elaborado pelos pesquisadores e formado por duas seções. As dez primeiras questões versavam sobre o perfil sociodemográfico e obstétrico das participantes, tendo sido investigadas as variáveis idade, cor/raça, religião, escolaridade, ocupação, renda familiar mensal, pessoas com quem residiam, situação conjugal, número de gestações/filhos e amamentação em gestações anteriores. A segunda parte, com seis questões subjetivas, explorou o objeto de estudo, focando em percepções, experiências e expectativas em torno do AME, abordadas por meio do diálogo.

No roteiro, as questões subjetivas foram assim redigidas: 1) “O que você sabe sobre o AME?”; 2) “Você acha que o AME é importante para seu filho? Por quê?”; 3) “Pretende amamentá-lo? Por quê? Se sim, como fará?”; 4) “Você conversa com outras pessoas da sua família (outras mulheres, companheiro) sobre o AME? Por quê? O que conversa?”; 5) “Durante as consultas de pré-natal, você recebeu alguma(s) orientação(ões) sobre o AME? Se sim, qual(is)? Como essa(s) orientação(ões) foi(ram) compartilhada(s)? Quem orientou?”; 6) “Você considera importante receber informações sobre o AME durante o pré-natal? Se sim, considera que essas informações influenciam sua decisão de amamentar? Por quê?”. Esse instrumento não foi submetido a teste piloto, mas permitiu apreender suficientemente o objeto, razão pela qual não se utilizou diário de campo, não foram incorporadas outras técnicas de coleta e não houve a necessidade de repetir entrevistas. Para não comprometer a espontaneidade dos depoimentos, optou-se por não compartilhar as transcrições com as participantes, para que pudessem conferi-las e referendá-las.

As entrevistas foram conduzidas pelos quatro estudantes de enfermagem, um homem e três mulheres, divididos em duplas e capacitados em reuniões de orientação, como atividades do PIBIC e reuniões de grupo de pesquisa vinculado à instituição dos autores, o qual desenvolve, sobretudo, pesquisas qualitativas. Como as entrevistas ocorreram em duas partes, correspondentes às seções do roteiro, a primeira parte foi registrada manualmente na versão impressa do instrumento. Apenas a segunda parte foi audiogravada no formato MP3, com duração média de 15 minutos, suficiente para alcançar o objetivo e compatível com outras pesquisas que utilizaram técnicas de análise qualitativa auxiliadas pelo mesmo *software* empregado neste estudo, as quais informaram duração média de 10 e 18 minutos^(16,17)^.

### Análise e Tratamento dos Dados

Os dados sociodemográficos e obstétricos foram tabulados no *Microsoft Office Excel* ^®^ (versão 2013) e analisados descritivamente para evidenciar os números absolutos e relativos. As entrevistas foram transcritas para formar um *corpus*, que foi importado para o *software Interface de R pour les Analyses Multidimensionnelles de Textes et de Questionnaires* (IRaMuTeQ^®^, versão 0.7, *alpha* 2) em arquivo único, sem formatação (arquivo do tipo .txt), com codificação de caracteres no padrão *Unicode Transformation Format 8-bit* (UTF 8), no intuito de realizar a análise lexical.

Em atendimento às especificidades técnicas e metodológicas desse *software* ^(18)^, os 20 textos que constituíram o *corpus* foram identificados individualmente por linhas de comando formadas pelos seguintes elementos: quatro asteriscos (****); espaço em branco; asterisco ao lado do código alfanumérico atribuído à participante; espaço em branco; nove variáveis sociodemográficas e obstétricas, identificadas por asterisco e códigos específicos (abreviação/sigla do nome da variável, *underline* e número ou abreviação/letra maiúscula/sigla complementar, de acordo com a natureza quantitativa ou qualitativa da variável), as quais foram separadas entre si por espaço em branco e definidas pelos pesquisadores como prioritárias, visando não alongar as linhas de comando mais que o necessário.

Essas variáveis e seus códigos foram: idade (“Id”, *underline* e número correspondente à idade da participante); cor/raça (“CR”, *underline* e abreviação da cor/raça, sendo “Br” para “branca”, “Pa” para “parda” e “Pr” para “preta”); escolaridade (“Esc”, *underline* e sigla da escolaridade, sendo “EFI” para “ensino fundamental incompleto”, “EMI” para “ensino médio incompleto”, “EMC” para “ensino médio completo”, “ET” para “ensino técnico”, “ESI” para “ensino superior incompleto” e “ESC” para “ensino superior completo”); religião (“Rel”, *underline* e inicial maiúscula da religião, sendo “C” para “católica”, “E” para “evangélica” e “N” para “sem religião”); situação conjugal (“SiC”, *underline* e abreviação ou inicial maiúscula da situação, sendo “Ca” para “casada”, “S” para “solteira” e “U” para “união consensual”); pessoas com quem residiam (“PQR”, *underline* e número de pessoas); renda familiar mensal (“RF”, *underline* e número correspondente à renda em salários mínimos, considerando que, no Brasil, em 2024, o salário vigente era de R$ 1.412,00, adicionando-se a palavra “menos” ou “mais”, quando necessário, para indicar que a renda era inferior ou superior ao número informado); gestações (“G”, *underline* e número); filhos (“F”, *underline* e número). Como exemplo dessa organização, para a primeira participante, atribuiu-se a linha de comando a seguir: **** *P1 *Id_29 *CR_Pa *Esc_EMC *Rel_E *SiC_S *PQR_2 *RF_1 *G_3 *F_2.

Utilizado frequentemente em pesquisas qualitativas e ancorado nas funcionalidades do *software* R, o IRaMuTeQ^®^ foi desenvolvido em 2009 pelo cientista francês Pierre Ratinaud, com cinco modalidades analíticas para assegurar a confiabilidade dos resultados: análise lexicográfica; especificidades e análise fatorial de correspondência; Classificação Hierárquica Descendente (CHD); análise de similitude; e nuvem de palavras^(19,20)^.

Neste estudo, empregou-se a CHD, por meio da qual o *corpus* foi desdobrado em segmentos de texto (STs) para gerar classes lexicais, segundo a complementariedade dos segmentos que as constituem, denotando significados que as individualizam, mas que, em conjunto, expressam o fenômeno estudado. Essas classes são ilustradas por palavras dispostas verticalmente em um dendrograma, às quais o IRaMuTeQ^®^ atribuiu, entre outros valores estatísticos, a frequência (F) de STs que apresentaram cada palavra em sua respectiva classe, e o qui-quadrado (X^2^), que demonstra a força associativa das palavras, considerando representativas aquelas que apresentaram p < 0,0001. Com essa modalidade, o *software* identifica a melhor possibilidade para formar classes que representem o conteúdo do *corpus*, resultando, muitas vezes, no aproveitamento de apenas uma parcela dos STs, a qual deve ser no mínimo de 75% para que a análise seja considerada confiável^(19,20)^.

As classes que derivaram da análise dos dados subjetivos foram organizadas em eixos temáticos, propiciando sua interpretação com base na literatura científica pertinente e atualizada. Esses eixos e as discussões a eles relacionadas compuseram um relatório encaminhado à instituição dos autores, que optaram por não o compartilhar com profissionais da unidade e gestantes, visando manter sua originalidade. Assim, não forneceram opiniões sobre os resultados, mas foi firmado o compromisso ético e técnico-científico de encaminhar o(s) artigo(s) aos profissionais tão logo seja(m) publicado(s) e, se necessário, agendar rodas de conversa para dialogar sobre os resultados, esclarecer dúvidas e discutir possíveis estratégias que permitam melhor intervir na realidade das gestantes.

### Aspectos Éticos

Atendeu-se à Resolução nº 466/2012 do Conselho Nacional de Saúde/Ministério da Saúde, obtendo-se a autorização da SESMA e a aprovação do Comitê de Ética em Pesquisa do Curso de Graduação em Enfermagem da Universidade do Estado do Pará, sob o Parecer nº 6.631.984, emitido em fevereiro de 2024. Todas as participantes assinaram o Termo de Consentimento Livre e Esclarecido. Para manter o sigilo das suas identidades, empregou-se um código alfanumérico constituído pela letra “P”, de “participante”, seguida por número cardinal, que indica a sequência das entrevistas. Ao longo dos resultados, para identificar os excertos, optou-se por destacar entre parênteses tanto os códigos alfanuméricos quanto as variáveis das linhas de comando.

## RESULTADOS

A idade das participantes variou de 19 a 37 anos, com média de 25,70 e predomínio da faixa etária de 19 a 23 (n = 9; 45%). No tocante à cor de pele, 17 (85%) declararam-se pardas, e quanto à religião, 11 (55%) eram evangélicas. Na escolaridade, 12 (60%) referiram ensino médio completo, e quanto à ocupação, 11 (55%) exerciam atividades remuneradas e nove (45%) realizavam somente atividades do lar. Na renda familiar mensal, dez (50%) declararam até um salário mínimo. Todas residiam com pelo menos uma pessoa, sendo que 12 (60%) eram casadas ou viviam em união consensual.

Em relação aos números de gestações e filhos, nove (45%) estavam na primeira gestação; seis (30%) estavam na segunda e tinham um filho; quatro (20%) estavam na terceira e tinham dois filhos; uma (5%) estava na quarta gestação e tinha três filhos; e 11 (55%) relataram ter amamentado em gestações anteriores.

O *corpus* intitulado “Aleitamento materno exclusivo: saberes de gestantes ribeirinhas na Atenção Primária à Saúde” foi desmembrado, resultando na divisão de seus 20 textos em 448 STs e na identificação de 15.674 ocorrências (formas ou palavras), com 2.149 palavras distintas e 1.132 hápax (palavras com frequência igual a um), representando 7,22% das ocorrências e 52,68% das palavras distintas. A média de palavras por ST foi de, aproximadamente, 34,99.

Por meio da CHD, foram aproveitados 342 STs (76,34%), estruturando dois *subcorpora*, com seis classes lexicais: o primeiro *subcorpus*, formado pelas classes 2 e 3, e o segundo, pelas classes 4, 6, 1 e 5, de acordo com a lógica de partição do *corpus* (Figura 1). Não foram considerados os dados subjetivos secundários que constituíram o percentual não aproveitado do *corpus* (23,66%).

**Figura 1:** Dendrograma da Classificação Hierárquica Descendente. Belém, Pará, Brasil, 2024.

Essas classes foram organizadas em dois eixos temáticos, associados respectivamente à construção social dos saberes sobre amamentação e aos saberes que repercutem na decisão de amamentar, motivo pelo qual os autores lhes atribuíram títulos que aludem a esses significados.

Compondo o primeiro eixo, as classes 3, 2, 1 e 5 foram denominadas “Importância das informações sobre amamentação no pré-natal”, “Orientações do enfermeiro sobre amamentação”, “Vivências e expectativas sobre o ato de amamentar” e “Diálogos com diferentes sujeitos no cotidiano”. No segundo eixo, as classes 4 e 6 receberam os títulos “Importância do aleitamento materno exclusivo para o crescimento e o desenvolvimento” e “Desafios e possibilidades da alimentação complementar”. Ambos são apresentados a seguir, destacando suas classes e alguns excertos emblemáticos.

### Eixo temático 1 – Construção social dos saberes sobre amamentação (classes 3, 2, 1 e 5)

Na classe 3, foram identificados 72 STs (21,05% do *corpus*) e 11 palavras representativas (p < 0,0001), indicando percepções que ressaltam a importância das orientações fornecidas por profissionais de saúde nas consultas de pré-natal (Figura 1).

Palavras como “informação” (X^2^ = 45,72), “receber” (X^2^ = 41,34), “orientação” (X^2^ = 41,17) e “consulta” (X^2^ = 28,01) representaram essa característica, indicando o compartilhamento de informações no ambiente formal da UMS. Nesse sentido, as participantes entendiam as orientações médicas e de enfermagem como ferramentas confiáveis e seguras, destacando que seu compartilhamento pode contribuir para melhor informá-las:

*Eu recebi essas informações antes de parir, pois a enfermeira fazia palestras com as gestantes. Foi quando adquiri um pouco de conhecimento e fiquei intrigada, porque a vovó falava uma coisa e a mamãe falava outra.* (P10, Id_22, CR_Pa, Esc_ET, Rel_E, SiC_U, PQR_2, RF_mais_1, G_2, F_1)

*Hoje foi a minha primeira consulta de pré-natal, então não recebi ainda informações sobre o aleitamento materno exclusivo. Porém, eu considero importante receber essas informações, inclusive na primeira consulta, nesse primeiro contato com os profissionais, porque fico informada, já que é algo que ainda não sei.* (P14, Id_22, CR_Pa, Esc_EMC, Rel_C, SiC_S, PQR_2, RF_1, G_1, F_0)

Formada por 39 STs (11,40% do *corpus*) e 11 palavras representativas (p < 0,0001), a classe 2 revelou como as orientações dos profissionais, sobretudo do enfermeiro, podem fortalecer a construção de saberes sobre amamentação. A palavra “enfermeiro” (X^2^ = 60,82), que apresentou o segundo maior qui-quadrado da classe, associada aos verbos “orientar” (X^2^ = 85,04) e “ensinar” (X^2^ = 32,79), expressa que as participantes reconheceram ter acesso, na atual gestação e/ou em gestação prévia, às orientações fornecidas por enfermeiros em diferentes serviços de saúde, como na UMS:

*A enfermeira orientava sobre o uso da pomada para assaduras e sobre chás, porque ela não era a favor de oferecer isso* [à criança]*. Ela dizia também sobre a importância da amamentação e que nem toda vez que a criança chorar era para amamentar. Tinha que aprender a entender qual era o horário de mamar e qual o horário que a criança estava com dor.* (P10, Id_22, CR_Pa, Esc_ET, Rel_E, SiC_U, PQR_2, RF_mais_1, G_2, F_1)

*Durante o pré-natal, a enfermeira sempre me orientava sobre como cuidar das mamas. Orientava a expor ao sol, praticar estímulos no mamilo durante o banho, não esfregar o sabonete no mamilo com muita frequência para não ressecar e, se necessário, ficar sem camisa para ter contato com a vitamina da luz do sol.* (P12, Id_29, CR_Br, Esc_EMC, Rel_E, SiC_U, PQR_3, RF_1, G_3, F_2)

Embora tenham declarado que foram orientadas no período gestacional, percebeu-se que as orientações não esclareceram suficientemente os aspectos que permeiam o tema. As palavras “mama” (X^2^ = 23,65) e “gestação” (X^2^ = 17,83), associadas aos verbos “realizar” (X^2^ = 46,95), “preparar” (X^2^ = 33,62) e “pegar” (X^2^ = 19,31), demonstram que, ainda assim, algumas participantes tinham vaga ideia de como preparar as mamas para o ato de amamentar, seja pela carência de orientações ou por certa imprecisão ou incoerência de alguns relatos:

*Tem que ter aconselhamento, pelo menos o básico, o que realmente não está sendo feito. Já fiz quatro consultas no pré-natal; faço perguntas para os profissionais de saúde, mas eles apenas falam que tem que hidratar as mamas, apenas isso.* (P9, Id_22, CR_Pa, Esc_ESI, Rel_N, SiC_Ca, PQR_1, RF_1, G_1, F_0)

*Durante as consultas de pré-natal, me perguntam se eu preparo a minha mama para amamentação. Dizem que é bom preparar, só que falo que eu não preparo, porque me orientaram no hospital que fazer isso não era bom.* (P15, Id_21, CR_Pa, Esc_EMI, Rel_E, SiC_U, PQR_2, RF_1, G_2, F_1)

Constituída por 62 STs (18,13% do *corpus*) e sete palavras representativas (p < 0,0001), a classe 1 caracterizou-se por palavras como “dor” (X^2^ = 20,95), “querer” (X^2^ = 16,06) e “experiência” (X^2^ = 15,86), abordando algumas repercussões da amamentação no cotidiano das gestantes que já vivenciaram esse ato como lactantes ou que tinham expectativa de amamentar. Identificou-se que a amamentação é um processo que pode gerar muitas expectativas e vivências, inclusive negativas, tendo em vista as sensações físicas e os afetos a ela relacionados.

Alguns STs demonstraram que o ato de amamentar pode gerar dor e incômodo nas mulheres, mesmo entre as que não são primíparas:

*Acredito que toda grávida passa por isso, mesmo as que não são primíparas. Até quem está no segundo parto vai sofrer a questão da dor na hora de amamentar.* (P11, Id_22, CR_Pa, Esc_ESI, Rel_E, SiC_S, PQR_11, RF_1, G_1, F_0)

*Muitas mulheres reclamam da amamentação; dizem que as mamas ficam flácidas e que sentem dor. Por exemplo, minha prima falou que sentiu muita dor, então fiquei assustada e isso me influencia, mas cada mulher tem sua experiência, cada mulher é* única*.* (P14, Id_22, CR_Pa, Esc_EMC, Rel_C, SiC_S, PQR_2, RF_1, G_1, F_0)

Em outros relatos, as participantes expressaram medo e incerteza, talvez por não conseguirem amamentar, com repercussões negativas no crescimento e desenvolvimento dos filhos:

*Depois de dez anos, ter que passar novamente por tudo aquilo* [dificuldades para amamentar o primeiro filho]*, apresento incerteza sobre amamentar.* (P4, Id_32, CR_Pa, Esc_EMC, Rel_E, SiC_Ca, PQR_2, RF_1, G_2, F_1)

*Tenho medo de criar expectativas sobre querer muito amamentar meu filho, e eu sei que pode acontecer de não conseguir. Então, tenho medo de me frustrar, de ficar decepcionada e não conseguir amamentar.* (P9, Id_22, CR_Pa, Esc_ESI, Rel_N, SiC_Ca, PQR_1, RF_1, G_1, F_0)

Apesar das sensações e dos afetos negativos, como destacado nos quatro excertos anteriores, identificou-se que a gestação também pode contribuir para formar vínculos entre mãe e filho, construídos ao longo das semanas de gestação e fortalecidos após o parto:

*Acredito que a amamentação exclusiva vai fazer eu criar um vínculo com meu bebê; vai existir uma ligação entre nós. Com o contato físico, ele vai sentir acolhimento através da amamentação.* (P6, Id_24, CR_Pr, Esc_EMC, Rel_N, SiC_Ca, PQR_2, RF_mais_1, G_1, F_0)

*Quando meu filho realizou a primeira pega* [referindo-se à primeira amamentação]*, eu chorei, porque tive uma sensação muito diferente, foi uma coisa que não sei explicar, uma força diferente de tudo nas mamas. Aconteceu uma coisa, um vínculo amoroso! Desde então, eu tive certeza de que teria que amamentar não por mim, mas por ele.* (P10, Id_22, CR_Pa, Esc_ET, Rel_E, SiC_U, PQR_2, RF_mais_1, G_2, F_1)

A classe 5 corresponde a 39 STs (11,40% do *corpus*), motivo pelo qual as classes 2 e 5 são as menores, com 15 palavras representativas (p < 0,0001), caracterizando-se por verbos como “parar” (X^2^ = 57,42), “deixar” (X^2^ = 33,41), “obrigar” (X^2^ = 31,44) e “olhar” (X^2^ = 23,51), além de outros termos, como “marido” (X^2^ = 25,49), “público” (X^2^ = 16,20) e “vergonha” (X^2^ = 16,20). Seu conteúdo aborda os diálogos que as gestantes desenvolveram sobre o tema com vários sujeitos sociais, como familiares, profissionais de saúde, vizinhos e até mesmo desconhecidos. Com base nos relatos, essas interações repercutiram significativamente nas percepções das mulheres, gerando insegurança em algumas delas, a depender das informações compartilhadas.

Alguns profissionais de saúde, que deveriam oferecer suporte e orientações adequadas, por vezes desaconselharam a amamentação e pressionaram por sua interrupção, tornando-a uma experiência estressante e insegura:

*As pessoas me pressionavam muito para parar de amamentar; falavam que o meu filho já estava grande. Uma vez, quando cheguei com ele doente na unidade* [de saúde]*, o médico que atendeu insistiu para eu parar de amamentar; falou que não tinha necessidade e que o bezerro apenas mama não sei até quantas semanas.* (P10, Id_22, CR_Pa, Esc_ET, Rel_E, SiC_U, PQR_2, RF_mais_1, G_2, F_1)

[...] *naquele momento, teve um embate; eu não iria deixar de amamentar meu filho somente porque uma pessoa estava incomodada, não sou obrigada a isso. É meu direito amamentar!* (P12, Id_29, CR_Br, Esc_EMC, Rel_E, SiC_U, PQR_3, RF_1, G_3, F_2)

Apesar de a amamentação ser um processo natural e essencial à vida, alguns relatos evidenciaram conflitos acerca da sua realização em espaços públicos, nos quais algumas pessoas, muitas vezes desconhecidas, alegaram desconforto ao presenciá-lo, imputando às mães certo constrangimento:

*As pessoas olhavam e diziam para o meu filho que ele estava grande, que deveria ter vergonha e parar de mamar.* Às *vezes, eu ficava constrangida, com vergonha e acuada.* (P10, Id_22, CR_Pa, Esc_ET, Rel_E, SiC_U, PQR_2, RF_mais_1, G_2, F_1)

*Tive situações de conflito com relação à amamentação. Me perguntaram se eu não tinha vergonha de amamentar em público; diziam que estavam incomodados em estar me observando amamentar e que eles não eram obrigados a olhar aquilo.* (P12, Id_29, CR_Br, Esc_EMC, Rel_E, SiC_U, PQR_3, RF_1, G_3, F_2)

Mesmo em face de diálogos desencorajadores, situações desrespeitosas e pressões sociais em certas ocasiões, inclusive por membros da família, as mulheres persistiram na amamentação ou declararam a intenção de amamentar, reconhecendo sua importância:

*Minha mãe falava que eu estava ficando feia* [por tanto amamentar] *e que o meu marido iria me trocar, mas isso é ele. Se não for para ser meu, não vai ser, mas eu não vou parar de amamentar.* (P10, Id_22, CR_Pa, Esc_ET, Rel_E, SiC_U, PQR_2, RF_mais_1, G_2, F_1)

*Meu marido diz que eu e ela* [cunhada] *somos parecidas, porque não temos vergonha. Se as pessoas falarem, eu não me importo; primeiro amamentarei meu filho. As outras pessoas que deixem de se importar.* (P16, Id_27, CR_Pa, Esc_EMI, Rel_C, SiC_S, PQR_4, RF_1, G_4, F_3)

### Eixo temático 2 – Saberes que repercutem na decisão de amamentar (classes 4 e 6)

Formada por 86 STs (25,15% do *corpus*), a classe 4 é a maior e apresenta 18 palavras representativas (p < 0,0001), como “desenvolvimento” (X^2^ = 59,89), “importante” (X^2^ = 39,64), “saúde” (X^2^ = 28,45), “crescer” (X^2^ = 27,51), “desenvolver” (X^2^ = 26,11), “leite materno” (X^2^ = 24,36), “alimentação” (X^2^ = 22,37), “benefício” (X^2^ = 19,39) e “vida” (X^2^ = 16,42). Associando-se às repercussões do AME no organismo das crianças, esses termos demonstram que as participantes caracterizaram a amamentação como uma prática que contribui para o crescimento e o desenvolvimento saudável de seus filhos, razão pela qual decidiam amamentar, ao relacionar o leite materno com o fortalecimento da imunidade da criança e a redução das possibilidades de adoecimento:

*Acredito que desenvolve tudo, as células, o crescimento, ter anticorpos para* [combater] *doenças e vírus. Acredito que a amamentação contribui de forma positiva para os meus dois filhos.* (P12, Id_29, CR_Br, Esc_EMC, Rel_E, SiC_U, PQR_3, RF_1, G_3, F_2)

*Os seis primeiros meses da amamentação são importantes para o desenvolvimento integral da criança, e isso vai ser bom para que ela, futuramente, não adquira doenças, tornando-a forte.* (P13, Id_37, CR_Pa, Esc_ESC, Rel_N, SiC_U, PQR_1, RF_mais_2, G_1, F_0)

Contudo, relataram que existem diferenças no leite materno e que algumas mulheres apresentam leite “mais fraco”, que não contribui para o crescimento e o desenvolvimento integral da criança, enquanto o leite “forte” é considerado mais nutritivo e responsável pelo crescimento e desenvolvimento adequados. Também destacaram que a produção de leite está relacionada à alimentação materna, pois acreditam que a alimentação é fonte de nutrientes, associada à quantidade e à qualidade do leite:

*Me falaram que devia ter uma alimentação saudável. Quanto mais me alimentava direito, mais leite materno iria produzir, porque, além de me cuidar e preparar, a gente dá uma vida melhor para eles* [os filhos]*.* (P1, Id_29, CR_Pa, Esc_EMC, Rel_E, SiC_S, PQR_2, RF_1, G_3, F_2)

*Acredito que* [o leite materno] é *muito importante, porque é a alimentação dele* [filho]*. Sei que a minha alimentação vai melhorar o leite e oferecer saúde para ele.* (P6, Id_24, CR_Pr, Esc_EMC, Rel_N, SiC_Ca, PQR_2, RF_mais_1, G_1, F_0)

Constituída por 44 STs (12,87% do *corpus*) e 15 palavras representativas (p < 0,0001), a classe 6 revela que as gestantes estavam cientes de que o AME deve ser realizado exclusivamente com leite materno durante os primeiros seis meses após o nascimento e, após esse período, outros alimentos devem ser oferecidos para complementar a amamentação. O termo “seis meses’’ (X^2^ = 56,60) apresentou o segundo maior qui-quadrado da classe, associando-se às palavras “completar” (X^2^ = 27,41) e “até” (X^2^ = 24,90):

*O aleitamento materno exclusivo é quando estou amamentando a criança até os seis meses. O médico fala que é sobre o bem-estar da criança. Ela não pode beber* água*, não pode comer e nem nada disso, porque pode adoecer.* (P3, Id_35, CR_Pa, Esc_EFI, Rel_E, SiC_S, PQR_2, RF_1, G_3, F_2)

*Não vou oferecer frutas para o meu filho de um mês, porque ele não vai saber comer uma maçã, não vai ter a prática da mastigação ou de engolir uma coisa que é mais rígida.* (P9, Id_22, CR_Pa, Esc_ESI, Rel_N, SiC_Ca, PQR_1, RF_1, G_1, F_0)

As palavras “água” (X^2^ = 76,22), “fruta” (X^2^ = 48,40), “chá” (X^2^ = 48,20), “comida” (X^2^ = 40,70), “mingau” (X^2^ = 38,30) e “suco” (X^2^ = 27,41) expressam que alguns fatores podem interferir na decisão de amamentar exclusivamente. Desafios relacionados aos aspectos culturais que permeiam a amamentação, como a crença de que o leite materno pode não saciar a criança, estimulavam o planejamento de outra fonte alimentar como alternativa. Além disso, a necessidade de retornar às atividades ocupacionais se destacou como aspecto socioeconômico relevante, pois algumas participantes apontaram que pode interferir no AME e prejudicar os vínculos entre mãe e filho, aumentando a possibilidade de introduzir outros alimentos precocemente. Esses aspectos foram indicados, por exemplo, nos depoimentos de P8 e P3, respectivamente:

*Vou oferecer mingau e leite materno. Se ele não ficar saciado com o leite, vou oferecer o mingau de manhã, pelo menos uma vez no dia.* (P8, Id_21, CR_Pa, Esc_EMC, Rel_C, SiC_U, PQR_3, RF_menos_1, G_1, F_0)

*Vou amamentar pelo menos quatro ou cinco meses. Não planejo amamentar até os seis meses, porque minha filha ficou muito apegada, não queria ficar com ninguém e eu não conseguia trabalhar.* (P3, Id_35, CR_Pa, Esc_EFI, Rel_E, SiC_S, PQR_2, RF_1, G_3, F_2)

## DISCUSSÃO

No primeiro eixo temático, a construção social dos saberes sobre amamentação evidenciou a importância do compartilhamento de informações sobre o AME, por meio de percepções das gestantes acerca das orientações que receberam dos profissionais de saúde, contexto no qual os diálogos com esses sujeitos apontaram para um processo de esclarecimento e fortalecimento dos saberes maternos, possibilitando inferir que as informações socialmente construídas repercutiam favoravelmente na decisão de amamentar. Contudo, durante o pré-natal, a comunicação insuficiente entre gestantes e profissionais pode gerar incertezas e conflitos relacionados a essa decisão.

No ciclo gravídico-puerperal, as orientações devem ser fundamentadas nas particularidades das gestantes, considerando aspectos culturais, econômicos, sociais e territoriais, visando favorecer uma gestação saudável e incentivar o AME. Essas mulheres necessitam de acolhimento e cuidados profissionais baseados na integralidade e longitudinalidade, além de processos educativos não meramente explicativos que valorizam suas inquietações. A APS é um cenário crucial nesse contexto, representando o principal nível de atenção acessado por gestantes e lactentes, em busca de informações e cuidados perinatais, sendo o espaço onde elas devem ser apoiadas para fortalecer seus saberes e monitorar a amamentação^(21,22)^.

Os profissionais que compartilham precocemente informações sobre o AME e informações complementares com gestantes e familiares atuam como agentes que promovem mecanismos protetores do aleitamento. O AME está diretamente relacionado à frequência das gestantes nas consultas de pré-natal, pois, quanto mais frequentam os serviços de saúde nesse período, maiores são as possibilidades de dialogar sobre o tema. Pesquisa realizada no estado da Bahia revelou iniquidades sociais e regionais como condições relevantes que interferem na qualidade dos cuidados perinatais, concluindo que gestantes da região Nordeste do Brasil ainda apresentam menor cobertura de serviços da APS, quando comparadas às da região Sul, que apresentam maiores indicadores de AME, possivelmente em virtude da melhor assistência que recebem na APS^(2)^.

As condições da assistência à saúde de gestantes ribeirinhas da região amazônica guardam similaridades com as de gestantes baianas, posto que, historicamente, as regiões Norte e Nordeste apresentam muitos desafios relacionados aos serviços de saúde e aspectos culturais peculiares, que devem ser considerados nas atividades assistenciais^(2,23)^. Constatou-se também a importância do enfermeiro, cujas orientações podem reforçar o ato de amamentar e consolidar os saberes das gestantes. Essas orientações materializaram-se nos esclarecimentos sobre as particularidades da amamentação, demonstrando as concepções prévias das participantes sobre o AME, embora os saberes de algumas estivessem tecnicamente mais estruturados e organizados que de outras.

A literatura aborda como as atividades de educação em saúde promovidas por enfermeiros podem impulsionar o conhecimento das gestantes, resultando no bom desempenho para amamentar, pois, com esclarecimentos e linguagem acessível, informações técnico-científicas podem ser compartilhadas de forma adequada, gerando conforto e segurança^(1,24)^. Explicações sobre os cuidados com as mamas, a posição corporal para que a criança seja amamentada e o tempo de amamentação, entre outros aspectos, podem influenciar positivamente o AME e impedir sua interrupção precoce^(25)^.

O enfermeiro é um agente ativo do cuidado que se aproxima dos usuários e cria vínculos, reconhecendo as características biopsicossociais de cada gestante como requisito necessário aos cuidados personalizados. Isso deve ocorrer mediante competências e habilidades construídas ao longo da formação acadêmica e do exercício profissional, com as quais realiza o exame clínico (anamnese e exame físico), incorporado à escuta sensível e às condutas necessárias para solucionar ou evitar problemas^(26)^.

Devido aos determinantes sociais de saúde das populações ribeirinhas e às falhas muitas vezes presentes nos serviços da APS, há entraves que limitam os cuidados personalizados, comprometendo sua efetividade. Por falta ou carência de profissionais qualificados, as condições de várias comunidades exigem esforços adicionais do enfermeiro para compartilhar informações sobre o AME e atender outras demandas^(5)^. Nesse sentido, o uso de tecnologias educacionais, com linguagem verbal e/ou não verbal acessível, pode facilitar orientações sobre o tema, desmistificando aspectos ultrapassados que possam interferir na decisão de amamentar^(25)^.

Na construção dos saberes, foram desveladas vivências e expectativas, motivo pelo qual, à luz dos resultados, entende-se que é fundamental conhecer as experiências maternas, pois, em certos casos, a amamentação pode gerar insegurança e repercutir negativamente no modo como as mulheres a representam, estimulando o desmame precoce^(27)^.

As preocupações que algumas expressaram quanto à possibilidade de não conseguir amamentar e à dor que o aleitamento pode causar nas mamas ocorreram pela falta de experiência, por experiências negativas ou pelo compartilhamento de saberes com outras mulheres. Fisiologicamente, as dores nas mamas resultam do ingurgitamento mamário ou de lesões nesses órgãos, tendo como principal causa o posicionamento corporal inadequado da criança ou da mãe ao amamentar, que pode interromper a produção de leite materno devido à redução dos estímulos de sucção, fragilizando as possibilidades de manter o aleitamento^(28)^. Apesar disso, as gestantes compreendiam a amamentação como processo que conecta seus protagonistas (mãe e criança), fortalecendo os vínculos entre eles. Infere-se que tal compreensão é capaz de motivar o AME, pois, na medida em que os saberes se estabelecem, há o encorajamento para que elas amamentem.

As percepções das gestantes foram expressivamente influenciadas por experiências de diálogo com sujeitos de seu cotidiano, como familiares, profissionais de saúde, vizinhos e outros. Em certas ocasiões, as interações sociais ocorreram de forma positiva e, em outras, de forma negativa, uma vez que possibilitaram vivenciar algumas situações de encorajamento para o AME, mas também atitudes desencorajadoras. À luz da literatura, esse fato pode repercutir nas dimensões biológica e psicossocial das gestantes^(29)^, pois uma parcela dos relatos demonstrou constrangimento e desmotivação para dar continuidade ao AME.

Por configurar um processo social, as redes de apoio são fundamentais para o aleitamento, pois as interações sociais influenciam fortemente sua execução e continuidade. Os saberes e as práticas sociais em torno do AME são heterogêneos e envoltos por essas interações, devido ao contexto social e ao papel desempenhado pela mulher na comunidade. Assim, o AME pode ocorrer com êxito ou não, a depender desse papel e de outros fatores, como experiências prévias, orientações acerca do tema e apoio familiar e social^(10,21)^. Nas relações familiares, há um processo histórico e sociocultural caracterizado pelo compartilhamento intergeracional de saberes sobre amamentação, encabeçado por sujeitos com notável representatividade, como mães e avós. Geralmente, há confiança nas orientações dos familiares, devido à convivência e compreensão de pertencimento por parte das gestantes^(21)^.

Quanto ao segundo eixo temático, os saberes que repercutem na decisão de amamentar revelaram o entendimento das gestantes sobre as repercussões do leite materno no organismo da criança, o qual foi desvelado por meio da associação simbólica do AME à figura de uma criança saudável e bem desenvolvida fisicamente. Também expressaram a ideia de que a alimentação da mãe influencia a produção de leite materno, na medida em que sua condição nutricional determina se esse alimento é suficiente ou insuficiente para atender às demandas nutricionais da criança.

Para além dos vínculos, o AME representa uma das principais formas de promover a saúde, oferecendo outros benefícios em curto ou longo prazo, pois tem efeitos importantes no desenvolvimento da musculatura bucal, visto que requer movimentos de sucção que envolvem a atividade simultânea de diferentes músculos. Também reduz a mortalidade infantil e a ocorrência de infecções, fenômenos alérgicos e obesidade, além de propiciar a liberação de ocitocina, hormônio que estimula sentimentos de prazer e bem-estar, atuando na secreção do leite materno, uma rica fonte de nutrientes e anticorpos, sendo o alimento mais completo e eficaz para nutrir o recém-nascido, pois fortalece seu sistema imunológico^(7,30)^.

Para a mãe, o ato de amamentar possibilita rápida recuperação no período pós-parto, diminuindo o risco de sangramento e a ocorrência de anemia, câncer de mama e câncer de ovário^(27,30)^. Destacam-se ainda seus benefícios para o desenvolvimento psíquico da criança, culminando por representar, junto a outros benefícios, uma alternativa sustentável para manter ou recuperar o equilíbrio da renda familiar^(8)^.

Um dos principais mitos do senso comum é a ideia do leite materno fraco e insuficiente para nutrir a criança. Faz-se tal ponderação considerando que, por sua completude, esse alimento dispõe de todos os compostos orgânicos e inorgânicos necessários à manutenção do organismo nos primeiros seis meses após o nascimento, como carboidratos, lipídios, proteínas, água e sais minerais^(5,31)^. Sua constituição é influenciada pela alimentação materna, razão pela qual é necessário cuidar da dieta para que seja produzido com melhor qualidade^(31)^. Nesse processo, os aspectos que mais devem se destacar são a adesão ao AME, o autocuidado e a autonomia da mulher durante sua execução^(21,29)^.

As gestantes expressaram a importância de amamentar de forma exclusiva nos primeiros seis meses e, após isso, introduzir outros alimentos de forma complementar. A literatura demonstra que alguns fatores podem reforçar esse entendimento e as práticas maternas a ele correspondentes, entre os quais destacam-se o acompanhamento regular do crescimento e do desenvolvimento, por meio das consultas de puericultura, especialmente nas unidades da APS, e as orientações fornecidas ainda no pré-natal, por profissionais qualificados e que garantam esclarecimento permanente, com informações sobre a suficiência nutricional do leite materno^(27,32)^.

Contudo, as participantes referiram algumas barreiras, as quais devem ser contornadas para que o AME seja efetivo, com ênfase nos aspectos culturais, visto que as crenças de terceiros, quando compartilhadas com as gestantes, podem influenciar sua decisão de amamentar. Inseridos no contexto ribeirinho, os determinantes culturais podem promover a disposição das mulheres para ofertar outros alimentos precocemente^(33)^, como evidenciado nos relatos de que certos alimentos provocam suposta saciedade quando comparados ao leite materno. Diante das vulnerabilidades sociais que circulam entre populações ribeirinhas, a escolaridade é um fator importante, pois repercute nessa decisão, haja vista que a introdução alimentar precoce tende a ser reduzida quando há devida compreensão sobre a importância do AME e da introdução de outros alimentos em momento oportuno^(34)^.

Também é válido ressaltar os aspectos socioeconômicos que, junto aos culturais, encorajaram precocemente a alimentação complementar, pois algumas mulheres intencionavam oferecer outros alimentos antes de completar o sexto mês, devido à necessidade de retornar às atividades ocupacionais. Dialogando com esse resultado, evidências demonstram que, quanto maior o nível socioeconômico das gestantes, menor é a chance de iniciar a alimentação complementar antes desse período^(34,35)^.

Os resultados evidenciaram, ainda, um conjunto de desafios relacionados ao AME que são inerentes a vários grupos humanos, pois são determinados socialmente por fatores como escolaridade e ocupação maternas, renda e apoio familiares, orientações e outros tipos de cuidado fornecidos pelos serviços de saúde, apontando fragilidades que devem ser superadas na atenção à saúde materno-infantil, no cotidiano desses grupos, como demonstrado em ampla revisão de escopo, que incluiu 145 estudos realizados na África, na Ásia, nas Américas e no Oriente Médio^(36)^.

Isso não invalida ou contradiz o fato de as populações ribeirinhas serem dotadas de especificidades que advêm da realidade que vivenciam e de seus modos de vida regidos pela dinâmica dos rios e das florestas, contexto no qual os processos de gestar, parir, tornar-se mãe e amamentar implicam muitos desafios coletivos, como reitera a literatura^(5,7,37,38)^. Todavia, considerando o AME como fenômeno social, é necessário que outros estudos sejam realizados com populações ribeirinhas em diferentes cenários para produzir evidências que apontem aspectos mais diretamente relacionados às especificidades dessas populações, marcando suas diferenças em relação às demais. Os resultados aqui desvelados podem subsidiar a concepção de novos objetos a serem investigados.

Como limitação deste estudo, menciona-se que foi realizado em uma unidade de saúde com gestantes ribeirinhas que vivem em um cenário específico no município de Belém, configurando menor representatividade geográfica dos dados. Por esse motivo, considera-se que os saberes acerca do tema foram influenciados por características culturais, socioeconômicas e territoriais, impedindo que sejam generalizados com maior amplitude, embora apresentem possíveis similaridades com as realidades de outras populações ribeirinhas nos cenários local, regional e nacional.

Assim, o estudo contribui com avanços para as áreas de enfermagem e saúde coletiva, pois pode fomentar espaços de discussão e reflexão entre estudantes e profissionais que atuam na assistência a essas populações, na gestão e no gerenciamento de serviços de saúde, no ensino e na pesquisa.

## CONCLUSÃO

Este estudo revelou os saberes de gestantes ribeirinhas sobre o AME na APS, demonstrando como influenciavam a decisão de amamentar. Informações qualificadas acerca do tema foram consideradas essenciais, sobretudo quando compartilhadas por enfermeiros e outros profissionais no pré-natal, diante de algumas informações insuficientes e que geravam insegurança e dúvidas entre as gestantes.

Apesar de vivências e expectativas desafiadoras e do desencorajamento suscitado por pessoas de seu convívio, as gestantes atribuíram à amamentação a oportunidade para fortalecer os vínculos com seus filhos. Também destacaram a importância do leite materno para o crescimento e o desenvolvimento da criança, ainda que paralelamente evidenciassem aspectos culturais e socioeconômicos que influenciavam a tomada de decisão.

Considerando a importância da APS para atender às necessidades de saúde dos grupos humanos, entende-se que tais resultados podem subsidiar estratégias para promover a saúde de populações ribeirinhas e incentivar o AME com informações culturalmente adequadas e práticas exequíveis, especialmente no contexto amazônico, valorizando a diversidade e as particularidades dessas populações.

Para isso, é preciso construir e implementar, na educação superior e na educação permanente, estratégias de ensino e aprendizagem que desenvolvam a competência cultural dos recursos humanos no campo da saúde, no intuito de induzir processos de formação alinhados às necessidades de grupos com características próprias e que demandam intervenções diferenciadas, como é o caso das populações ribeirinhas. Faz-se tal ponderação para ressaltar que a universalidade, a integralidade e a equidade, princípios doutrinários do Sistema Único de Saúde brasileiro, devem ser efetivadas nas práticas de atenção.

Estratégias que permitam esse desenvolvimento são particularmente necessárias no cotidiano dos profissionais de enfermagem, sobretudo dos enfermeiros, cujos processos de trabalho inerentes às práticas de cuidado, gestão, ensino, pesquisa e participação política demandam alto senso de responsabilidade científica, ética, técnica e social, para que o exercício consciente e seguro da profissão mobilize transformações em meio aos desafios dos cenários onde habitam populações ribeirinhas.

## Data Availability

Os dados que dão suporte a este estudo podem ser disponibilizados mediante solicitação ao autor correspondente.

# Translation: Conocimientos de gestantes ribereñas sobre la lactancia materna exclusiva en el contexto de la Atención Primaria de Salud

**DOI:** 10.1590/1980-220X-REEUSP-2024-0361es

**Authors:** Gabriela Tayane Santana do Espirito Santo, Jennifer Hillary Costa da Conceição Pompeu, Paulo Daniel Pereira Raad, Julia Oliveira Mendes, Erlon Gabriel Rego de Andrade, Eliene do Socorro da Silva Santos, Laura Maria Vidal Nogueira, Ivaneide Leal Ataíde Rodrigues

**Affiliations:** 1 Universidade do Estado do Pará, Escola de Enfermagem Magalhães Barata, Belém, PA, Brasil.; 2 Universidade do Estado do Pará, Escola de Enfermagem Magalhães Barata, Programa de Pós-Graduação em Enfermagem, Belém, PA, Brasil.

**Keywords:** Lactancia Materna, Personas Embarazadas, Población Rural, Salud Rural, Atención Primaria de Salud

## Abstract

**Objetivo:**

Discutir el conocimiento de mujeres ribereñas embarazadas sobre la lactancia materna exclusiva y su influencia en la decisión de amamantar.

**Método:**

Estudio descriptivo y cualitativo, realizado con 20 gestantes de la Unidad Municipal de Salud de Ilha de Cotijuba, en Belém, Pará, Brasil. Los datos fueron recolectados a través de entrevistas individuales semiestructuradas, realizadas entre marzo y junio de 2024. El *corpus* fue sometido a análisis léxico con el *software Interface de R pour les Analyses Multidimensionnelles de Textes et de Questionnaires* (0.7, *alpha* 2), utilizando Clasificación Jerárquica Descendente.

**Resultados:**

Se identificaron 448 segmentos textuales, de los cuales se utilizaron 342 (76,34%), generándose seis clases léxicas, organizadas en dos ejes temáticos, que presentaron la construcción de conocimientos sobre lactancia materna, mediados por las relaciones sociales, y los conocimientos que impactan la decisión de amamantar, resaltando la importancia de la lactancia materna para el crecimiento y desarrollo de recién nacidos y lactantes y la interfaz con la alimentación complementaria.

**Conclusión:**

A pesar de las experiencias y expectativas desafiantes y del desánimo suscitado por las personas de su entorno, las mujeres embarazadas asumieron la lactancia materna como una oportunidad para fortalecer los vínculos con sus hijos.

## INTRODUCCIÓN

La lactancia materna exclusiva (LME) es el suministro exclusivo de leche materna a recién nacidos y lactantes, que puede producirse a través del pecho o bebiendo leche ordeñada, sin que se añada ningún otro tipo de alimento a la dieta. Debe fomentarse hasta el sexto mes después del nacimiento, y se recomienda mantenerla de forma complementaria hasta que el niño cumpla dos años. Además de promover una nutrición adecuada, con repercusiones en el crecimiento y desarrollo de todos los sistemas orgánicos y reducir la mortalidad infantil, la LME refuerza el vínculo entre madre e hijo y contribuye a preservar o aumentar la salud materna^(1,2)^.

Teniendo en cuenta los beneficios de la leche materna, la Organización Mundial de la Salud (OMS) espera que en 2030 al menos el 70% de los niños reciban lactancia materna exclusiva a la edad adecuada^(3)^. En Brasil, aunque los indicadores de lactancia materna hayan aumentado en las últimas décadas, la prevalencia de LME entre los niños menores de seis meses era de sólo el 45,8%, según datos del Estudio Nacional de Alimentación y Nutrición Infantil (ENANI), publicado en 2021^(4)^.

Independientemente de su lejanía de los centros urbanos, las comunidades tradicionales, como las poblaciones ribereñas, aún se encuentran en situación de vulnerabilidad, caracterizada, entre otros factores, por bajos niveles de escolaridad y falta de atención a la salud. En ese contexto sociogeográfico, regido por la dinámica de ríos y bosques, las gestantes suelen tener dificultades para acceder a los servicios de Atención Primaria de Salud (APS), como las consultas médicas y de enfermería prenatales, debido a las peculiaridades territoriales y económicas que dificultan su desplazamiento a las unidades de salud^(5,6)^.

Se sabe que el control prenatal es un escenario propicio para la consolidación de ideas y decisiones relacionadas con la LME, incluso entre las gestantes ribereñas, pues es en ese período que ellas se enteran de las diversas exigencias que atraviesan los procesos de embarazo, parto y maternidad, así como de la importancia de la LME^(7,8)^.

Las poblaciones cuyas principales actividades económicas y de subsistencia son la agricultura y la pesca adoptan dietas relacionadas con estas actividades^(9)^. El conocimiento tradicional, compartido entre generaciones, puede ser eficaz para fomentar la lactancia materna, pero también puede generar o reforzar mitos sobre el tema, hasta el punto de interferir en la decisión de amamantar, favorecer el destete precoz y la inclusión de otros alimentos antes del periodo recomendado^(10)^.

Al investigar el conocimiento de esas gestantes sobre la lactancia materna, es posible comprender los aspectos culturales, económicos y sociales que interfieren en esa práctica y, así, reflexionar sobre cómo esos aspectos pueden vincularse a las políticas públicas y a los programas de salud que buscan atender las demandas sanitarias.

Dada la relevancia del tema y la necesidad de compartirlo, se elaboró la siguiente pregunta orientadora: ¿cuál es el conocimiento de las gestantes ribereñas sobre la LME y cómo influye en su decisión de amamantar? Para responder a esta pregunta, este estudio se propuso analizar el conocimiento de las gestantes ribereñas sobre la LME y su influencia en su decisión de amamantar.

## MÉTODO

### Tipo de Estudio

Es un estudio descriptivo y cualitativo, guiado por los *criterios COnsolidados para REportar la Investigación Cualitativa* (COREQ)^(11)^. Optamos por asociar el carácter descriptivo a este enfoque, considerando las posibilidades que presenta para caracterizar fenómenos, incluidos los sociales, prestando atención a los datos subjetivos, pero también a los aspectos que particularizan a determinados grupos en relación con estos fenómenos^(12)^.

### Sitio del Estudio

El estudio fue realizado en la Unidad Municipal de Salud (UMS) de la Isla de Cotijuba, localizada en el Distrito Administrativo de Outeiro, en el municipio de Belém, Pará, Brasil. Vinculada al Departamento Municipal de Salud de Belém (SESMA), esta unidad ofrece servicios típicos de APS, como consultas prenatales.

La isla tiene cerca de 10.000 habitantes y, a pesar de las transformaciones urbanas resultantes de factores como su proximidad a otros distritos administrativos de Belém y la intensificación de las actividades turísticas, todavía muestra debilidades en la provisión de políticas públicas, como es el caso de la atención sanitaria local^(13,14)^. Se eligió este escenario porque sus características geográficas y socioculturales se adecuaban al interés en investigar el fenómeno de la LME en el contexto de una población ribereña.

### Población y Criterios de Selección

Participaron 20 gestantes ribereñas mayores de 18 años, independientemente de la edad gestacional y del número de embarazos anteriores, inscritas y en control prenatal regular en la UMS. Se estableció una edad mínima de 18 años, teniendo en cuenta que el embarazo en menores puede implicar aspectos legales o riesgo de deterioro emocional y psicológico para la gestante. Se optó por excluir a aquellas con deterioro físico y/o cognitivo que imposibilitara entrevistarlas. Sin embargo, no hubo exclusiones ni retiradas; sólo dos se negaron a participar por no querer ser entrevistadas después de tres intentos.

Durante el periodo de recolección de datos, 22 mujeres embarazadas fueron elegibles y participaron el 90,91% (20/22). Esto coincide con la recomendación de la bibliografía, que indica que entre 20 y 30 participantes es un número adecuado para alcanzar los objetivos de una investigación con un enfoque cualitativo^(15)^.

### Recolección de Datos

Inicialmente, los investigadores visitaron la UMS para presentarse a los profesionales y darles a conocer el objetivo y los procedimientos de la investigación, momento en el que conocieron la dinámica de la atención prenatal y reservaron una habitación con el gestor para realizar las actividades. Las gestantes fueron seleccionadas por conveniencia y abordadas personalmente, de forma individual, antes o después de sus consultas, cuando fueron invitadas a participar. Para garantizar comodidad y privacidad, las que aceptaron participar fueron llevadas a una sala privada, donde sólo estaban presentes la participante y los entrevistadores, que presentaron y aclararon los detalles de la investigación en lenguaje sencillo, con el objetivo de obtener la aceptación formal.

Cabe destacar que los profesionales de la unidad y las embarazadas no conocían de antemano a los investigadores y, por lo tanto, desconocían su formación académica y profesional, así como sus aspiraciones. Por lo tanto, decidimos aclarar todos los aspectos necesarios al personal y a las gestantes, incluyendo el hecho de que la investigación formaba parte de un conjunto de actividades extracurriculares desarrolladas por cuatro estudiantes de enfermería del Programa Institucional de Becas de Iniciación Científica (PIBIC), dos de ellas becarios y dos voluntarios.

La Recolección de datos se realizó entre marzo y junio de 2024 a través de entrevistas individuales semiestructuradas, utilizando un guión elaborado por las investigadoras y compuesto por dos secciones. Las diez primeras preguntas se referían al perfil sociodemográfico y obstétrico de las participantes, investigando las variables edad, color/raza, religión, escolaridad, ocupación, renta familiar mensual, personas con las que vivían, estado civil, número de embarazos/hijos y lactancia en embarazos anteriores. La segunda parte, con seis preguntas subjetivas, exploró el objeto de estudio, centrándose en las percepciones, experiencias y expectativas en torno a la LME, abordadas a través del diálogo.

En el guión, las preguntas subjetivas estaban redactadas del siguiente modo: 1) «¿Qué sabe sobre la LME?»; 2) «¿Cree que la LME es importante para su hijo? ¿Por qué?»; 3) «¿Quiere amamantar a su hijo? ¿Por qué? Si es así, ¿cómo lo hará?»; 4) «¿Habla con otras personas de su familia (otras mujeres, su pareja) sobre la LME? ¿Por qué? ¿De qué hablan?»; 5) «Durante sus consultas prenatales, ¿recibió algún consejo sobre lactancia materna? En caso afirmativo, ¿cuáles? ¿Cómo se compartieron estas instrucciones? ¿Quién dio los consejos?»; 6) «¿Considera importante recibir información sobre la LME durante la atención prenatal? En caso afirmativo, ¿cree que esta información influyó en su decisión de dar el pecho? ¿Por qué?». Este instrumento no se sometió a una prueba piloto, pero permitía comprender suficientemente el tema, por lo que no se utilizó un diario de campo, no se incorporaron otras técnicas de recogida y no hubo necesidad de repetir las entrevistas. Para no poner en peligro la espontaneidad de los testimonios, se decidió no compartir las transcripciones con las participantes para que pudieran comprobarlas y consultarlas.

Las entrevistas fueron realizadas por cuatro estudiantes de enfermería, un hombre y tres mujeres, divididos en parejas y formados en reuniones de orientación, como las actividades del PIBIC y las reuniones de un grupo de investigación vinculado a la institución de los autores, que realiza principalmente investigación cualitativa. Como las entrevistas se realizaron en dos partes, correspondientes a las secciones del guión, la primera parte se grabó manualmente en la versión impresa del instrumento. Solamente la segunda parte fue audio-grabada en formato MP3, con una duración media de 15 minutos, suficiente para alcanzar el objetivo y compatible con otros estudios que utilizaron técnicas de análisis cualitativo auxiliadas por el mismo *software* utilizado en este estudio, que reportaron duraciones medias de 10 y 18 minutos^(16,17)^.

### Análisis y Procesamiento de Los Datos

Los datos sociodemográficos y obstétricos fueron tabulados en *Microsoft Office Excel* ^®^ (versión 2013) y analizados descriptivamente para mostrar números absolutos y relativos. Las entrevistas se transcribieron para formar un *corpus*, que se importó al *programa informático Interface de R pour les Analyses Multidimensionnelles de Textes et de Questionnaires* (IRaMuTeQ^®^, versión 0.7, *alfa* 2) como archivo único sin formato (archivo .txt), con codificación de caracteres en el estándar *Unicode Transformation Format 8-bit* (UTF 8), para realizar el análisis léxico.

De acuerdo con las especificidades técnicas y metodológicas de este *programa informático* ^(18)^, los 20 textos que componían el *corpus* se identificaron individualmente mediante líneas de comando compuestas por los siguientes elementos: cuatro asteriscos (****); espacio en blanco; asterisco junto al código alfanumérico asignado al participante; espacio en blanco; nueve variables sociodemográficas y obstétricas, identificadas por asterisco y códigos específicos (abreviatura/abreviatura del nombre de la variable, *subrayado* y número o abreviatura/letra mayúscula/abreviatura complementaria, según la naturaleza cuantitativa o cualitativa de la variable), que se separaron entre sí por espacio en blanco y fueron definidas por los investigadores como prioritarias, para no alargar las líneas de comando más de lo necesario.

Estas variables y sus códigos eran edad («Id», *subrayado* y número correspondiente a la edad del participante); color/raza («CR», *subrayado* y abreviatura de color/raza, «Br» para «blanco», «Pa» para «moreno» y «Pr» para «negro»); escolaridad («Esc», *subrayado* y abreviatura de escolaridad, «EFI» para «educación primaria incompleta», «EMI» para «educación secundaria incompleta», «EMC» para «educación secundaria completa», «ET» para «educación técnica», «ESI» para «educación superior incompleta» y «ESC» para «educación superior completa»); religión («Rel», *subrayado* e inicial mayúscula de la religión, donde «C» para «católica», «E» para «evangélica» y «N» para «sin religión»); estado civil («SiC», *subrayado* y abreviatura o inicial mayúscula del estado, donde «Ca» para «casado», «S» para «soltero» y «U» para «unión consensual»); personas con las que vivían («PQR», *subrayado* y número de personas); renta familiar mensual («RF», *subrayado* y número correspondiente a la renta en salarios mínimos, considerando que en Brasil, en 2024, el salario vigente era de R$ 1.412,00, añadiendo la palabra «menos» o «más» cuando fuera necesario para indicar que la renta era inferior o superior a la cifra indicada); embarazos («G», *subrayado* y número); hijos («F», *subrayado* y número). Como ejemplo de esta organización, se dio la siguiente línea de comandos al primer participante: **** *P1 *Id_29 *CR_Pa *Esc_EMC *Rel_E *SiC_S *PQR_2 *RF_1 *G_3 *F_2.

Frecuentemente utilizado en la investigación cualitativa y anclado en las funcionalidades del *software* R, el IRaMuTeQ^®^ fue desarrollado en 2009 por el científico francés Pierre Ratinaud, con cinco modalidades analíticas para garantizar la fiabilidad de los resultados: análisis lexicográfico; análisis factorial de especificidades y correspondencias; Clasificación Jerárquica Descendente (CJD); análisis de similitud; y nube de palabras^(19,20)^.

En este estudio se utilizó la CJD, mediante la cual se descompuso el *corpus* en segmentos textuales (STs) para generar clases léxicas, según la complementariedad de los segmentos que los componen, denotando significados que los individualizan, pero que en conjunto expresan el fenómeno estudiado. Estas clases se ilustran mediante palabras dispuestas verticalmente en un dendrograma, al que el IRaMuTeQ^®^ asignó, entre otros valores estadísticos, la frecuencia (F) de STs que presentaban cada palabra en su respectiva clase, y el chi-cuadrado (X^2^), que muestra la fuerza asociativa de las palabras, considerando representativas aquellas con p < 0,0001. Con esta modalidad, el *software* identifica la mejor posibilidad de formar clases que representen el contenido del *corpus*, resultando muchas veces en la utilización de apenas una parte de las STs, que debe ser de por lo menos 75% para que el análisis sea considerado confiable^(19,20)^.

Las clases derivadas del análisis de los datos subjetivos se organizaron en ejes temáticos, lo que permitió interpretarlas a partir de la literatura científica pertinente y actualizada. Esos ejes y las discusiones relacionadas con ellos constituyeron un informe enviado a la institución por las autoras, que optaron por no compartirlo con los profesionales de la unidad ni con las gestantes para mantener su originalidad. Como tal, no opinaron sobre los resultados, pero asumieron el compromiso ético y técnico-científico de remitir el/los artículo/s a los profesionales tan pronto como fuera publicado y, si fuera necesario, programar círculos de conversación para discutir los resultados, aclarar dudas y discutir posibles estrategias para intervenir mejor en la realidad de las gestantes.

### Aspectos Éticos

El estudio cumplió la Resolución 466/2012 del Consejo Nacional de Salud/Ministerio de Salud, obteniendo autorización del SESMA y aprobación del Comité de Ética en Investigación del Curso de Pregrado en Enfermería de la *Universidade do Estado do Pará*, bajo el Dictamen nº 6.631.984, emitido en febrero de 2024. Todos los participantes firmaron el consentimiento informado. Para mantener la confidencialidad de sus identidades, se utilizó un código alfanumérico compuesto por la letra «P», de «participante», seguida de un número cardinal, que indica la secuencia de las entrevistas. A lo largo de los resultados, para identificar los extractos, se decidió destacar entre paréntesis tanto los códigos alfanuméricos como las variables de las líneas de comando.

## RESULTADOS

La edad de los participantes osciló entre 19 y 37 años, con una media de 25,70 años y un predominio del grupo de 19 a 23 años (n = 9; 45%). En cuanto al color de la piel, 17 (85%) dijeron ser morenos, y en cuanto a la religión, 11 (55%) eran evangélicos. En cuanto a la escolaridad, 12 (60%) habían terminado el bachillerato, y en cuanto a la ocupación, 11 (55%) tenían un trabajo remunerado y nueve (45%) sólo realizaban tareas domésticas. En cuanto a los ingresos familiares mensuales, diez (50%) declararon tener hasta un salario mínimo. Todas vivían con al menos otra persona, y 12 (60%) estaban casadas o en unión libre.

Con relación al número de embarazos e hijos, nueve (45%) estaban en su primer embarazo; seis (30%) estaban en el segundo y tenían un hijo; cuatro (20%) estaban en el tercero y tenían dos hijos; una (5%) estaba en su cuarto embarazo y tenía tres hijos; y 11 (55%) informaron haber amamantado en embarazos anteriores.

El *corpus* titulado «Lactancia materna exclusiva: conocimiento de las gestantes ribereñas en Atención Primaria de Salud» fue desglosado, resultando en la división de sus 20 textos en 448 STs y en la identificación de 15.674 ocurrencias (formas o palabras), con 2.149 palabras distintas y 1.132 hápax (palabras con frecuencia igual a uno), representando 7,22% de las ocurrencias y 52,68% de las palabras distintas. El número medio de palabras por ST era de aproximadamente 34,99.

A través de la CJD, se utilizaron 342 STs (76,34%), estructurando dos *subcorpus* con seis clases léxicas: el primer *subcorpus*, formado por las clases 2 y 3, y el segundo, por las clases 4, 6, 1 y 5, según la lógica de partición del *corpus* (Figura 1). No se tuvieron en cuenta los datos subjetivos secundarios que constituían el porcentaje no utilizado del *corpus* (23,66%).

**Figura 1:** Dendrograma de clasificación jerárquica descendente. Belém, Pará, Brasil, 2024.

Estas clases se organizaron en dos ejes temáticos, asociados respectivamente a la construcción social del conocimiento sobre lactancia materna y al conocimiento que afecta la decisión de amamantar, razón por la cual las autoras les dieron títulos que aluden a estos significados.

Componiendo el primer eje, las clases 3, 2, 1 y 5 se denominaron «Importancia de la información sobre la lactancia materna durante la atención prenatal», «Orientación de la enfermera sobre la lactancia materna», «Experiencias y expectativas sobre la lactancia materna» y «Diálogos con diferentes sujetos en la vida cotidiana». En el segundo eje, las clases 4 y 6 se titularon «Importancia de la lactancia materna exclusiva para el crecimiento y el desarrollo» y «Retos y posibilidades de la alimentación complementaria». Ambas se presentan a continuación, destacando sus clases y algunos extractos emblemáticos.

### Eje temático 1 – Construcción social del conocimiento sobre la lactancia materna (clases 3, 2, 1 y 5)

En la clase 3, se identificaron 72 STs (21,05% del *corpus*) y 11 palabras representativas (p < 0,0001), indicando percepciones que enfatizan la importancia de la orientación proporcionada por los profesionales de salud durante las consultas prenatales (Figura 1).

Palabras como «información» (X^2^ = 45,72), «recibir» (X^2^ = 41,34), «orientación» (X^2^ = 41,17) y «consulta» (X^2^ = 28,01) representaron esta característica, indicando el intercambio de información en el ambiente formal de la UMS. En este sentido, las participantes percibieron la orientación médica y de enfermería como herramientas fiables y seguras, destacando que compartirla podría ayudar a informarlas mejor:

*Recibí esta información antes de dar a luz, porque la enfermera daba charlas a las embarazadas. Fue entonces cuando adquirí un poco de conocimiento y me quedé intrigada, porque la abuela decía una cosa y mamá otra.* (P10, Id_22, CR_Pa, Esc_ET, Rel_E, SiC_U, PQR_2, RF_mais_1, G_2, F_1)

*Hoy ha sido mi primera cita prenatal, así que aún no he recibido ninguna información sobre la lactancia materna exclusiva. Sin embargo, creo que es importante recibir esta información, incluso en la primera cita, en este primer contacto con los profesionales, porque estoy informada, ya que es algo que todavía no sé.* (P14, Id_22, CR_Pa, Esc_EMC, Rel_C, SiC_S, PQR_2, RF_1, G_1, F_0)

Compuesta por 39 STs (11,40% del *corpus*) y 11 palabras representativas (p < 0,0001), la clase 2 reveló cómo la orientación por parte de profesionales, especialmente enfermeras, puede reforzar la construcción del conocimiento sobre la lactancia materna. La palabra «enfermero» (X^2^ = 60,82), que tuvo el segundo chi-cuadrado más alto de la clase, asociada a los verbos «orientar» (X^2^ = 85,04) y «enseñar» (X^2^ = 32,79), expresa que las participantes reconocieron que tuvieron acceso, durante su embarazo actual y/o embarazos anteriores, a orientaciones proporcionadas por enfermeras en diferentes servicios de salud, como la UMS:

*La enfermera me orientó sobre el uso de pomada para la dermatitis del pañal y sobre los tés, porque ella no era partidaria de ofrecer eso* [al niño]*. También me habló de la importancia de la lactancia materna y de que no siempre que el niño lloraba era para darle el pecho. Había que aprender a entender cuándo era el momento de dar el pecho y cuándo el niño tenía dolor.* (P10, Id_22, CR_Pa, Esc_ET, Rel_E, SiC_U, PQR_2, RF_mais_1, G_2, F_1)

*Durante los cuidados prenatales, la enfermera siempre me daba consejos sobre cómo cuidar mis pechos. Me dijo que los expusiera al sol, que estimulara el pezón mientras me duchaba, que no me frotara el pezón con jabón con demasiada frecuencia para que no se resecara y que, si era necesario, fuera sin camiseta para que pudiera tener contacto con la vitamina de la luz solar.* (P12, Id_29, CR_Br, Esc_EMC, Rel_E, SiC_U, PQR_3, RF_1, G_3, F_2)

Aunque afirmaron haber recibido orientaciones durante el embarazo, se observó que éstas no aclaraban suficientemente los aspectos que impregnan el tema. Las palabras «mama» (X^2^ = 23,65) y «embarazo» (X^2^ = 17,83), asociadas a los verbos «realizar» (X^2^ = 46,95), «preparar» (X^2^ = 33,62) y «tomar» (X^2^ = 19,31), muestran que, aun así, algunas de las participantes tenían una idea vaga de cómo preparar sus mamas para el acto de amamantar, ya sea por falta de orientación o por cierta imprecisión o incoherencia en algunos de los relatos:

*Tiene que haber asesoramiento, al menos lo básico, que no se está haciendo realmente. He ido a cuatro citas prenatales; hago preguntas a los profesionales sanitarios, pero sólo me dicen que hay que hidratar los pechos, eso es todo.* (P9, Id_22, CR_Pa, Esc_ESI, Rel_N, SiC_Ca, PQR_1, RF_1, G_1, F_0)

*Durante mis citas prenatales, me preguntan si preparo mis pechos para la lactancia. Dicen que es bueno prepararlo, pero yo digo que no lo preparo, porque en el hospital me dijeron que no era bueno hacerlo.* (P15, Id_21, CR_Pa, Esc_EMI, Rel_E, SiC_U, PQR_2, RF_1, G_2, F_1)

Compuesta por 62 STs (18,13% del *corpus*) y siete palabras representativas (p < 0,0001), la clase 1 se caracterizó por palabras como «dolor» (X^2^ = 20,95), «querer» (X^2^ = 16,06) y «experiencia» (X^2^ = 15,86), abordando algunas de las repercusiones de la lactancia en la vida cotidiana de las gestantes que ya habían experimentado este acto como amamantadoras o que esperaban amamantar. Se identificó que el amamantamiento es un proceso que puede generar muchas expectativas y vivencias, incluso negativas, dadas las sensaciones físicas y afectos relacionados a él.

Algunos STs mostraron que el acto de amamantar puede generar dolor y malestar en las mujeres, incluso entre las que no son primíparas:

*Creo que todas las mujeres embarazadas pasan por esto, incluso las que no son primíparas. Incluso las que están en su segundo parto experimentarán dolor al amamantar.* (P11, Id_22, CR_Pa, Esc_ESI, Rel_E, SiC_S, PQR_11, RF_1, G_1, F_0)

*Muchas mujeres se quejan de la lactancia; dicen que se les caen los pechos y sienten dolor. Por ejemplo, mi prima dijo que sentía mucho dolor, así que me asusté y eso me influye, pero cada mujer tiene su propia experiencia, cada mujer es* única*.* (P14, Id_22, CR_Pa, Esc_EMC, Rel_C, SiC_S, PQR_2, RF_1, G_1, F_0)

En otros informes, las participantes expresaron miedo e incertidumbre, tal vez por no haber podido amamantar, con repercusiones negativas para el crecimiento y el desarrollo de sus hijos:

*Después de diez años, tener que volver a pasar por todo eso* [dificultades para amamantar al primer hijo]*, me siento insegura sobre la lactancia materna.* (P4, Id_32, CR_Pa, Esc_EMC, Rel_E, SiC_Ca, PQR_2, RF_1, G_2, F_1)

*Tengo miedo de crearme expectativas por querer amamantar mucho a mi hijo, y sé que puede ocurrir que no lo consiga. Así que tengo miedo de frustrarme, decepcionarme y no poder amamantar.* (P9, Id_22, CR_Pa, Esc_ESI, Rel_N, SiC_Ca, PQR_1, RF_1, G_1, F_0)

A pesar de los sentimientos y afectos negativos, como se destaca en los cuatro extractos anteriores, se identificó que el embarazo también puede contribuir a la formación de vínculos entre madre e hijo, construidos a lo largo de las semanas de gestación y fortalecidos después del parto:

*Creo que la lactancia materna exclusiva me hará crear un vínculo con mi bebé; habrá una conexión entre nosotros. Con el contacto físico, se sentirá acogido a través de la lactancia materna.* (P6, Id_24, CR_Pr, Esc_EMC, Rel_N, SiC_Ca, PQR_2, RF_mais_1, G_1, F_0)

*Cuando mi hijo se prendió por primera* vez [refiriéndose a su primera lactancia]*, lloré porque tenía una sensación muy diferente, algo que no puedo explicar, una fuerza distinta a todo lo que había en mis pechos. Algo sucedió, ¡un vínculo amoroso! Desde entonces, estaba segura de que tenía que dar el pecho no por mí, sino por* él*.* (P10, Id_22, CR_Pa, Esc_ET, Rel_E, SiC_U, PQR_2, RF_mais_1, G_2, F_1)

La clase 5 corresponde a 39 STs (11,40% del *corpus*), por lo que las clases 2 y 5 son las más pequeñas, con 15 palabras representativas (p < 0,0001), caracterizadas por verbos como «parar» (X^2^ = 57,42), «dejar» (X^2^ = 33,41), «obligar» (X^2^ = 31,44) y «mirar» (X^2^ = 23,51), además de otros términos como «marido» (X^2^ = 25,49), «público» (X^2^ = 16,20) y «vergüenza» (X^2^ = 16,20). Su contenido abarca los diálogos que las embarazadas mantuvieron sobre el tema con diversos sujetos sociales, como familiares, profesionales sanitarios, vecinos e incluso desconocidos. De acuerdo con los relatos, estas interacciones tuvieron un impacto significativo en las percepciones de las mujeres, generando inseguridad en algunas de ellas, dependiendo de la información compartida.

Algunos profesionales sanitarios, que se suponía que debían ofrecer apoyo y orientación adecuados, a veces desaconsejaban la lactancia y presionaban a las mujeres para que la dejaran, convirtiéndola en una experiencia estresante e insegura:

*La gente me presionaba mucho para que dejara de dar el pecho; decían que mi hijo ya era grande. Una vez, cuando llegué a la unidad* [de salud] *con* él *enfermo, el médico que me atendió insistió en que dejara de amamantarlo; dijo que no era necesario y que el ternero sólo mama durante no sé cuántas semanas.* (P10, Id_22, CR_Pa, Esc_ET, Rel_E, SiC_U, PQR_2, RF_mais_1, G_2, F_1)

[...] *en ese momento, hubo un choque; yo no iba a dejar de amamantar a mi hijo sólo porque alguien se molestara, no estoy obligada a hacerlo. Tengo derecho a dar el pecho.* (P12, Id_29, CR_Br, Esc_EMC, Rel_E, SiC_U, PQR_3, RF_1, G_3, F_2)

A pesar del hecho de que la lactancia materna es un proceso natural y esencial para la vida, algunos informes mostraron conflictos sobre la lactancia materna en espacios públicos, donde algunas personas, a menudo extraños, alegaron incomodidad al presenciarla, causando a las madres cierta vergüenza:

*La gente miraba a mi hijo y le decía que era demasiado grande, que debería avergonzarse y dejar de darle el pecho. A veces sentía vergüenza, pudor y miedo.* (P10, Id_22, CR_Pa, Esc_ET, Rel_E, SiC_U, PQR_2, RF_mais_1, G_2, F_1)

*He tenido situaciones conflictivas en relación con la lactancia materna. Me preguntaron si me avergonzaba dar el pecho en público; dijeron que se sentían incómodos viéndome dar el pecho y que no estaban obligados a mirarlo.* (P12, Id_29, CR_Br, Esc_EMC, Rel_E, SiC_U, PQR_3, RF_1, G_3, F_2)

Incluso ante diálogos desalentadores, situaciones irrespetuosas y presión social en determinadas ocasiones, incluso por parte de familiares, las mujeres persistieron en la lactancia o declararon su intención de hacerlo, reconociendo su importancia:

*Mi madre decía que me estaba poniendo fea* [de tanto dar el pecho] *y que mi marido me cambiaría, pero eso es* cosa suya. *Si no es mío, no lo será, pero no voy a dejar de dar el pecho.* (P10, Id_22, CR_Pa, Esc_ET, Rel_E, SiC_U, PQR_2, RF_mais_1, G_2, F_1)

*Mi marido dice que ella* [cuñada] *y yo nos parecemos porque no tenemos vergüenza. Si la gente habla, no me importa; primero le daré el pecho a mi hijo. Que los demás dejen de preocuparse.* (P16, Id_27, CR_Pa, Esc_EMI, Rel_C, SiC_S, PQR_4, RF_1, G_4, F_3)

### Eje temático 2 – Conocimientos que afectan a la decisión de amamantar (clases 4 y 6)

Compuesta por 86 STs (25,15% del *corpus*), la clase 4 es la más numerosa y tiene 18 palabras representativas (p < 0,0001), como «desarrollo» (X^2^ = 59,89), «importante» (X^2^ = 39,64), «salud» (X^2^ = 28,45), «crecer» (X^2^ = 27,51), «desarrollar» (X^2^ = 26,11), «leche materna» (X^2^ = 24,36), «alimentación» (X^2^ = 22,37), «beneficio» (X^2^ = 19,39) y «vida» (X^2^ = 16,42). Cuando se asocian a las repercusiones de la lactancia en el cuerpo de los niños, estos términos muestran que las participantes caracterizan la lactancia como una práctica que contribuye al crecimiento y desarrollo saludable de sus hijos, razón por la que decidieron amamantarlos, relacionando la leche materna con el fortalecimiento de la inmunidad del niño y la reducción de las posibilidades de enfermedad:

*Creo que lo desarrolla todo, las células, el crecimiento, tener anticuerpos para* [combatir] *enfermedades y virus. Creo que la lactancia materna contribuye positivamente a mis dos hijos.* (P12, Id_29, CR_Br, Esc_EMC, Rel_E, SiC_U, PQR_3, RF_1, G_3, F_2)

*Los seis primeros meses de lactancia son importantes para el desarrollo integral del niño, y esto será bueno para que no contraiga enfermedades en el futuro, haciéndolo fuerte.* (P13, Id_37, CR_Pa, Esc_ESC, Rel_N, SiC_U, PQR_1, RF_mais_2, G_1, F_0)

Sin embargo, informaron de que existen diferencias en la leche materna y que algunas mujeres tienen una leche «más débil», que no contribuye al pleno crecimiento y desarrollo del niño, mientras que la leche «fuerte» se considera más nutritiva y responsable de un crecimiento y desarrollo adecuados. También hicieron hincapié en que la producción de leche está relacionada con la nutrición materna, ya que creen que la nutrición es una fuente de nutrientes, asociada a la cantidad y la calidad de la leche:

*Me dijeron que debía seguir una dieta sana. Cuanto más comiera bien, más leche materna produciría, porque además de cuidarme y prepararme, les damos* [a los niños] *una vida mejor.* (P1, Id_29, CR_Pa, Esc_EMC, Rel_E, SiC_S, PQR_2, RF_1, G_3, F_2)

*Creo que* [la leche materna] *es muy importante, porque es su* [del niño] *alimento. Sé que mi dieta mejorará la leche y le hará más sano.* (P6, Id_24, CR_Pr, Esc_EMC, Rel_N, SiC_Ca, PQR_2, RF_mais_1, G_1, F_0)

Compuesta por 44 STs (12,87% del *corpus*) y 15 palabras representativas (p < 0,0001), la clase 6 revela que las mujeres embarazadas eran conscientes de que la lactancia materna debe llevarse a cabo exclusivamente con leche materna durante los seis primeros meses después del nacimiento y, después de este periodo, deben ofrecerse otros alimentos para complementar la lactancia materna. El término «seis meses» (X^2^ = 56,60) tuvo el segundo chi-cuadrado de clase más alto, asociado a las palabras «completar» (X^2^ = 27,41) y «hasta» (X^2^ = 24,90):

*La lactancia materna exclusiva es cuando amamanto al niño hasta los seis meses. El médico dice que se trata del bienestar del niño. No pueden beber agua, no pueden comer ni nada de eso, porque podrían ponerse enfermos.* (P3, Id_35, CR_Pa, Esc_EFI, Rel_E, SiC_S, PQR_2, RF_1, G_3, F_2)

*No voy a ofrecer fruta a mi bebé de un mes porque no sabrá comer una manzana, no tendrá la práctica de masticar o tragar algo que es más rígido.* (P9, Id_22, CR_Pa, Esc_ESI, Rel_N, SiC_Ca, PQR_1, RF_1, G_1, F_0)

Las palabras «agua» (X^2^ = 76,22), «fruta» (X^2^ = 48,40), «té» (X^2^ = 48,20), «comida» (X^2^ = 40,70), «papilla» (X^2^ = 38,30) y «jugo» (X^2^ = 27,41) expresan que algunos factores pueden interferir en la decisión de amamantar exclusivamente. Los desafíos relacionados con los aspectos culturales que impregnan la lactancia materna, como la creencia de que la leche materna puede no satisfacer al niño, fomentaron la planificación de otra fuente de alimentación como alternativa. Además, la necesidad de reincorporarse a las actividades laborales destacó como un aspecto socioeconómico relevante, ya que algunas participantes señalaron que podría interferir con la lactancia y poner en peligro el vínculo entre madre e hijo, aumentando la posibilidad de introducir otros alimentos de forma precoz. Estos aspectos se indicaron, por ejemplo, en las declaraciones de P8 y P3, respectivamente:

*Le ofrezco papilla y leche materna. Si no toma suficiente leche, le ofreceré papilla por la mañana, al menos una vez al día.* (P8, Id_21, CR_Pa, Esc_EMC, Rel_C, SiC_U, PQR_3, RF_menos_1, G_1, F_0)

*Voy a dar el pecho durante al menos cuatro o cinco meses. No pienso dar el pecho hasta los seis meses, porque mi hija se encariñó mucho, no quería estar con nadie y yo no podía trabajar.* (P3, Id_35, CR_Pa, Esc_EFI, Rel_E, SiC_S, PQR_2, RF_1, G_3, F_2)

## DISCUSIÓN

En el primer eje temático, la construcción social del conocimiento sobre lactancia materna destacó la importancia de compartir información sobre la LME, a través de las percepciones de las gestantes sobre la orientación que recibieron de los profesionales de salud, contexto en el que los diálogos con estos sujetos apuntaron a un proceso de clarificación y fortalecimiento del conocimiento materno, permitiendo inferir que la información socialmente construida tuvo un impacto favorable en la decisión de amamantar. Sin embargo, durante la atención prenatal, una comunicación insuficiente entre las gestantes y los profesionales puede generar incertidumbres y conflictos relacionados con esta decisión.

En el ciclo embarazo-puerperio, la orientación debe basarse en las particularidades de las mujeres embarazadas, considerando aspectos culturales, económicos, sociales y territoriales, con el objetivo de favorecer un embarazo saludable y fomentar la lactancia materna. Estas mujeres necesitan una atención profesional basada en la integralidad y la longitudinalidad, así como procesos educativos que no sean meramente explicativos y valoren sus inquietudes. La APS es un escenario crucial en este contexto, representando el principal nivel de atención al que acceden las gestantes y lactantes en busca de información y cuidados perinatales, y el espacio donde deben ser apoyadas para reforzar sus conocimientos y realizar el seguimiento de la lactancia materna^(21,22)^.

Los profesionales que comparten información temprana sobre la LME e información complementaria con las gestantes y sus familias actúan como agentes que promueven mecanismos protectores de la lactancia materna. La LME está directamente relacionada con la asistencia de las gestantes a las consultas prenatales, ya que cuanto más acuden a los servicios de salud durante este periodo, mayores son las posibilidades de dialogar sobre el tema. Un estudio realizado en el estado de Bahía reveló las desigualdades sociales y regionales como condiciones relevantes que interfieren en la calidad de la atención perinatal, concluyendo que las gestantes de la región Nordeste de Brasil aún tienen menor cobertura de los servicios de APS en comparación con las del Sur, que presentan mayores indicadores de AME, posiblemente debido a la mejor atención que reciben en la APS^(2)^.

Las condiciones de atención a la salud de las gestantes ribereñas de la región amazónica son similares a las de las gestantes de Bahía, dado que las regiones Norte y Nordeste históricamente enfrentaron muchos desafíos relacionados con los servicios de salud y aspectos culturales peculiares, que deben ser tenidos en cuenta en las actividades de atención^(2,23)^. Señalamos también la importancia de las enfermeras, cuya orientación puede reforzar el acto de amamantar y consolidar los conocimientos de las gestantes. Esa orientación se materializó en aclaraciones sobre las particularidades de la lactancia materna, demostrando las concepciones previas de las participantes sobre la LME, aunque algunos de sus conocimientos fueran técnicamente más estructurados y organizados que otros.

La literatura discute como las actividades de educación para la salud promovidas por enfermeros pueden potenciar los conocimientos de las gestantes, resultando en un buen desempeño de la lactancia, pues con aclaraciones y lenguaje accesible, las informaciones técnico-científicas pueden ser compartidas de forma adecuada, generando comodidad y seguridad^(1,24)^. Las explicaciones sobre el cuidado del pecho, la posición del cuerpo para el niño a ser amamantado y el tiempo de amamantamiento, entre otros aspectos, pueden influir positivamente en la lactancia y prevenir su interrupción precoz^(25)^.

Las enfermeras son agentes activos del cuidado que se acercan a las usuarias y crean vínculos, reconociendo las características biopsicosociales de cada gestante como requisito necesario para una atención personalizada. Esto debe ocurrir a través de las habilidades y destrezas construidas durante su formación académica y práctica profesional, con las que realizan el examen clínico (anamnesis y exploración física), incorporando la escucha sensible y el comportamiento necesario para resolver o evitar problemas^(26)^.

Debido a los determinantes sociales de la salud de las poblaciones ribereñas y a las deficiencias frecuentemente presentes en los servicios de APS, existen obstáculos que limitan la atención personalizada, comprometiendo su eficacia. Debido a la falta o escasez de profesionales cualificados, las condiciones de muchas comunidades requieren esfuerzos adicionales por parte de las enfermeras para compartir información sobre la AME y atender otras demandas^(5)^. En este sentido, el uso de tecnologías educativas, con lenguaje verbal y/o no verbal accesible, puede facilitar la orientación sobre el tema, desmitificando aspectos obsoletos que pueden interferir en la decisión de amamantar^(25)^.

En la construcción del conocimiento, se develaron experiencias y expectativas, por lo que, a la luz de los resultados, se entiende que es fundamental conocer las vivencias maternas, ya que, en ciertos casos, la lactancia puede generar inseguridad e impactar negativamente en la forma en que las mujeres se la representan, incentivando el destete precoz^(27)^.

Las preocupaciones que algunas mujeres expresaron sobre la posibilidad de no poder amamantar y el dolor que la lactancia puede causar se debieron a la falta de experiencia, a experiencias negativas o a compartir conocimientos con otras mujeres. Fisiológicamente, el dolor mamario es consecuencia de la congestión mamaria o de lesiones en estos órganos, cuya causa principal es la posición corporal inadecuada del niño o de la madre al amamantar, que puede interrumpir la producción de leche materna debido a la reducción de los estímulos de succión, lo que debilita las posibilidades de mantener la lactancia^(28)^. A pesar de eso, las gestantes entendieron el amamantamiento como un proceso que conecta a sus protagonistas (madre e hijo), fortaleciendo los lazos entre ellos. Se infiere que esta comprensión es capaz de motivar la lactancia porque, a medida que se establece el conocimiento, se animan a amamantar.

Las percepciones de las embarazadas se vieron significativamente influidas por las experiencias de diálogo con personas de su vida cotidiana, como familiares, profesionales sanitarios, vecinos y otros. En algunas ocasiones, las interacciones sociales fueron positivas y en otras negativas, ya que permitieron experimentar algunas situaciones alentadoras para la lactancia, pero también actitudes desalentadoras. A la luz de la literatura, esto puede repercutir en la dimensión biológica y psicosocial de las gestantes^(29)^, ya que algunos de los relatos mostraron vergüenza y desmotivación para continuar con la lactancia.

Como proceso social, las redes de apoyo son fundamentales para la lactancia materna, ya que las interacciones sociales influyen mucho en su puesta en práctica y continuidad. Los conocimientos y las prácticas sociales en torno a la LME son heterogéneos y están rodeados de estas interacciones, debido al contexto social y al papel que desempeñan las mujeres en la comunidad. Así, la LME puede ser exitosa o no, dependiendo de este rol y de otros factores como experiencias previas, orientación sobre el tema y apoyo familiar y social^(10,21)^. En las relaciones familiares, existe un proceso histórico y sociocultural caracterizado por el intercambio intergeneracional de conocimientos sobre lactancia materna, liderado por personas con notable representatividad, como madres y abuelas. Generalmente, existe confianza en la orientación proporcionada por los familiares, debido a la convivencia y comprensión de pertenencia por parte de las gestantes^(21)^.

En cuanto al segundo eje temático, el conocimiento que incide en la decisión de amamantar reveló la comprensión de las gestantes sobre las repercusiones de la leche materna en el cuerpo del niño, que se reveló a través de la asociación simbólica de la lactancia con la figura de un niño sano y bien desarrollado físicamente. También expresaron la idea de que la dieta de la madre influye en la producción de leche materna, hasta el punto de que su estado nutricional determina si este alimento es suficiente o insuficiente para satisfacer las demandas nutricionales del niño.

Además del vínculo afectivo, la lactancia materna es una de las principales vías de promoción de la salud, ofreciendo otros beneficios a corto o largo plazo, ya que tiene importantes efectos sobre el desarrollo de la musculatura bucal, al requerir movimientos de succión que implican la actividad simultánea de diferentes músculos. También reduce la mortalidad infantil y la ocurrencia de infecciones, fenómenos alérgicos y obesidad, además de promover la liberación de oxitocina, hormona que estimula la sensación de placer y bienestar, actuando en la secreción de leche materna, rica fuente de nutrientes y anticuerpos, siendo el alimento más completo y eficaz para la nutrición del recién nacido, ya que fortalece su sistema inmunológico^(7,30)^.

Para la madre, el acto de amamantar le permite recuperarse rápidamente en el posparto, reduciendo el riesgo de hemorragias y la aparición de anemia, cáncer de mama y cáncer de ovario^(27,30)^. También son destacables sus beneficios para el desarrollo psicológico del niño, culminando en el hecho de que, junto con otros beneficios, representa una alternativa sostenible para mantener o recuperar una renta familiar equilibrada^(8)^.

Uno de los principales mitos del sentido común es la idea de que la leche materna es débil e insuficiente para nutrir al niño. Esto se basa en el hecho de que, por ser completo, este alimento contiene todos los compuestos orgánicos e inorgánicos necesarios para el mantenimiento del organismo en los primeros seis meses después del nacimiento, como carbohidratos, lípidos, proteínas, agua y sales minerales^(5,31)^. Su constitución está influenciada por la nutrición materna, por lo que es necesario cuidar la dieta para que se produzca con mejor calidad^(31)^. En este proceso, los aspectos en los que más se debe incidir son la adherencia a la LME, el autocuidado y la autonomía de la mujer durante su realización^(21,29)^.

Las gestantes manifestaron la importancia de la lactancia materna exclusiva durante los primeros seis meses y, después, introducir otros alimentos complementarios. La literatura muestra que algunos factores pueden reforzar esta comprensión y las prácticas maternas correspondientes, incluyendo el monitoreo regular del crecimiento y desarrollo a través de las consultas de puericultura, especialmente en las unidades de APS, y la orientación proporcionada durante la atención prenatal por profesionales calificados que garantizan la aclaración continua, con información sobre la suficiencia nutricional de la leche materna^(27,32)^.

Sin embargo, las participantes mencionaron algunas barreras que deben ser superadas para que la lactancia materna sea efectiva, con énfasis en los aspectos culturales, ya que las creencias de los demás, cuando son compartidas con las gestantes, pueden influir en su decisión de amamantar. Insertados en el contexto ribereño, los determinantes culturales pueden promover la disposición de las mujeres a ofrecer otros alimentos precozmente^(33)^, como demuestran los relatos de que ciertos alimentos causan supuesta saciedad cuando comparados a la leche materna. Teniendo en cuenta las vulnerabilidades sociales que circulan entre las poblaciones ribereñas, la escolarización es un factor importante, ya que repercute en esta decisión, dado que la introducción precoz de alimentos tiende a reducirse cuando existe una comprensión adecuada de la importancia de la LME y de la introducción de otros alimentos en el momento adecuado^(34)^.

También cabe destacar los aspectos socioeconómicos que, junto con los culturales, incentivaron la alimentación complementaria precoz, ya que algunas mujeres pretendían ofrecer otros alimentos antes del sexto mes debido a la necesidad de retomar sus actividades ocupacionales. En diálogo con este resultado, la evidencia muestra que cuanto mayor es el nivel socioeconómico de las gestantes, menor es la probabilidad de que inicien la alimentación complementaria antes de este período^(34,35)^.

Los resultados también mostraron un conjunto de desafíos relacionados con la alimentación complementaria que son inherentes a diversos grupos humanos, ya que están socialmente determinados por factores como la educación y la ocupación de la madre, los ingresos y el apoyo de la familia, la orientación y otros tipos de atención prestada por los servicios de salud, señalando las deficiencias que deben superarse en la atención de la salud maternoinfantil en la vida cotidiana de estos grupos, como se demuestra en una amplia revisión de alcance que incluyó 145 estudios realizados en África, Asia, las Américas y Oriente Medio^(36)^.

Esto no invalida ni contradice el hecho de que las poblaciones ribereñas estén dotadas de especificidades derivadas de la realidad que viven y de sus modos de vida regidos por la dinámica de ríos y bosques, contexto en el que los procesos de gestar, parir, ser madre y amamantar implican muchos desafíos colectivos, como reitera la literatura^(5,7,37,38)^. Sin embargo, considerando que la LME es un fenómeno social, es necesario realizar otros estudios con poblaciones ribereñas en diferentes escenarios para producir evidencias que apunten a aspectos más directamente relacionados con las especificidades de esas poblaciones, marcando sus diferencias en relación a otras. Los resultados aquí descubiertos pueden subsidiar la concepción de nuevos objetos a ser investigados.

Una limitación de este estudio es que fue realizado en una unidad de salud con gestantes ribereñas que viven en un ambiente específico del municipio de Belém, lo que hace que los datos sean menos representativos geográficamente. Por este motivo, se considera que el conocimiento sobre el tema estuvo influenciado por características culturales, socioeconómicas y territoriales, impidiendo una generalización más amplia, aunque muestra posibles semejanzas con las realidades de otras poblaciones ribereñas en los escenarios local, regional y nacional.

Así, el estudio contribuye para avances en las áreas de enfermería y salud pública, ya que puede incentivar la discusión y reflexión entre estudiantes y profesionales que actúan en el cuidado de esas poblaciones, en la gestión de servicios de salud, en la enseñanza y en la investigación.

## CONCLUSIÓN

Este estudio reveló el conocimiento de las gestantes ribereñas sobre la lactancia materna en APS, mostrando cómo influyó en su decisión de amamantar. La información calificada sobre el tema fue considerada esencial, especialmente cuando compartida por enfermeros y otros profesionales durante el control prenatal, frente a algunas informaciones insuficientes que generaron inseguridad y dudas entre las gestantes.

A pesar de las difíciles experiencias y expectativas y del desánimo de su entorno, las embarazadas consideraron la lactancia materna como una oportunidad para reforzar los vínculos con sus hijos. También destacaron la importancia de la leche materna para el crecimiento y desarrollo del niño, aunque al mismo tiempo resaltaron aspectos culturales y socioeconómicos que influyeron en su toma de decisiones.

Considerando la importancia de la APS en la atención a las necesidades de salud de los grupos humanos, se entiende que estos resultados pueden subsidiar estrategias para promover la salud de las poblaciones ribereñas y estimular la lactancia materna con informaciones culturalmente apropiadas y prácticas viables, especialmente en el contexto amazónico, valorizando la diversidad y las particularidades de estas poblaciones.

Para ello, es necesario construir e implementar estrategias de enseñanza y aprendizaje en la educación superior y en la educación continua que desarrollen la competencia cultural de los recursos humanos en el campo de la salud, con el objetivo de inducir procesos de formación alineados con las necesidades de grupos con características propias y que requieren intervenciones diferenciadas, como es el caso de las poblaciones ribereñas. Se trata de enfatizar que la universalidad, la integralidad y la equidad, principios doctrinarios del Sistema Único de Salud brasileño, deben ser implementados en las prácticas asistenciales.

Estrategias que posibiliten ese desarrollo son particularmente necesarias en el cotidiano de los profesionales de enfermería, en especial de las enfermeras, cuyos procesos de trabajo inherentes al cuidado, gestión, enseñanza, investigación y participación política exigen un alto sentido de responsabilidad científica, ética, técnica y social, para que el ejercicio consciente y seguro de la profesión pueda movilizar transformaciones en medio a los desafíos de los escenarios donde viven las poblaciones ribereñas.

## Data Availability

Los datos que respaldan este estudio están disponibles a solicitud del autor correspondiente.
